# Soil pH, developmental stages and geographical origin differently influence the root metabolomic diversity and root-related microbial diversity of *Echium vulgare* from native habitats

**DOI:** 10.3389/fpls.2024.1369754

**Published:** 2024-06-24

**Authors:** Cintia Csorba, Nebojša Rodić, Livio Antonielli, Angela Sessitsch, Angeliki Vlachou, Muhammad Ahmad, Stéphane Compant, Markus Puschenreiter, Eva M. Molin, Andreana N. Assimopoulou, Günter Brader

**Affiliations:** ^1^ AIT Austrian Institute of Technology GmbH, Center for Health & Bioresources, Bioresources Unit, Tulln, Austria; ^2^ Aristotle University of Thessaloniki, School of Chemical Engineering, Laboratory of Organic Chemistry and Center for Interdisciplinary Research and Innovation, Natural Products Research Centre of Excellence (NatPro-AUTh), Thessaloniki, Greece; ^3^ Department of Forest Growth, Silviculture and Genetics, Austrian Research Centre for Forests (BFW), Vienna, Austria; ^4^ Institute of Soil Research, University of Natural Resources and Life Sciences Vienna, Vienna, Austria

**Keywords:** metabolome, microbiome, soil, plant, *Boraginaceae*, *Echium*, multiomics, environment

## Abstract

Improved understanding of the complex interaction between plant metabolism, environmental conditions and the plant-associated microbiome requires an interdisciplinary approach: Our hypothesis in our multiomics study posited that several environmental and biotic factors have modulating effects on the microbiome and metabolome of the roots of wild *Echium vulgare* plants. Furthermore, we postulated reciprocal interactions between the root metabolome and microbiome. We investigated the metabolic content, the genetic variability, and the prokaryotic microbiome in the root systems of wild *E. vulgare* plants at rosette and flowering stages across six distinct locations. We incorporated the assessment of soil microbiomes and the measurement of selected soil chemical composition factors. Two distinct genetic clusters were determined based on microsatellite analysis without a consistent alignment with the geographical proximity between the locations. The microbial diversity of both the roots of *E. vulgare* and the surrounding bulk soil exhibited significant divergence across locations, varying soil pH characteristics, and within the identified plant genetic clusters. Notably, acidophilic bacteria were characteristic inhabitants of both soil and roots under acidic soil conditions, emphasizing the close interconnectedness between these compartments. The metabolome of *E. vulgare* significantly differed between root samples from different developmental stages, geographical locations, and soil pH levels. The developmental stage was the dominant driver of metabolome changes, with significantly higher concentrations of sugars, pyrrolizidine alkaloids, and some of their precursors in rosette stage plant roots. Our study featured the complex dynamics between soil pH, plant development, geographical locations, plant genetics, plant metabolome and microbiome, shedding light on existing knowledge gaps.

## Introduction

The plant family *Boraginaceae* comprise several species with medicinal properties, such as *Alkanna tinctoria* L. Tausch (Papageorgiou et al., 2008; Tappeiner et al., 2014), *Lithospermum* sp (Hisa et al., 1998; Yazaki, 2017; Khosravi et al., 2019), *Symphytum officinale* L (Trifan et al., 2020), *Pulmonaria officinalis* L (Krzyżanowska-Kowalczyk et al., 2018), or *Echium vulgare* L. (Viper´s bugloss (Wang et al., 2022). The latter species, our study subject, is a biennial or hapaxanth flowering herb native to Europe and a rich source of several secondary metabolites (Eruygur et al., 2016). Among these metabolites, pyrrolizidine alkaloids (PAs) (Lucchetti et al., 2016) are toxic, but also medicinally relevant bioactive secondary metabolites, such as the naphtoquinones alkannin/shikonin and their derivatives (A/S) can be found, similar to *A. tinctoria* (Papageorgiou et al., 1999; Eruygur et al., 2016). A/S are enantiomeric constituents and are of cosmeceutical and pharmaceutical interest (Papageorgiou et al., 2008). Toxic PAs of *Echium* plants pose direct danger to grazing livestock (Wiedenfeld and Edgar, 2011) as well as humans through *Echium*-derived honey (Beales et al., 2007). Former studies on PAs in *E. vulgare* were limited to foliage (Skoneczny et al., 2015), pollen (Kast et al., 2018), and floral nectar (Lucchetti et al., 2016), in the present study we focused on the lesser studied roots.

The quality and quantity of primary and secondary metabolites of plants are shaped by different parameters and are highly interconnected with the plant’s microbiome, e.g. the rhizosphere microbial communities thriving at the root-soil interface by root exudates (Kawasaki et al., 2016; Zhalnina et al., 2018), and the endosphere or root inhabiting microbiota by the root metabolism (Pang et al., 2021a). Plants are known to actively recruit beneficial microorganisms from the surrounding soil through numerous mechanisms as a response to various environmental signals, such as pathogen presence (Liu et al., 2021; Sui et al., 2023) or through the signaling of already present endophytes (Ujvári et al., 2021). Plant-associated microbial communities are further influenced by the plant genotype (Brown et al., 2020; Cordovez et al., 2021) and plant developmental stage (Yuan et al., 2015; Cordovez et al., 2021). The plant genotype and in- or between population genetic differences may lead to different levels of plant metabolites (Cadot et al., 2021). Furthermore, the plant developmental stage has been found to be the main factor of altering root secondary metabolic activity in *Boraginaceae* such as *E. vulgare* (Skoneczny et al., 2017). Soil, with its specific chemical, physical and biological characteristics, has been identified as a primary driver of plant-associated microbiome composition and plant metabolism both in above- and below-ground plant organs (ZarraonaIndia et al., 2015; Aguirre-Becerra et al., 2021; Cadot et al., 2021). The highly plastic metabolic activity of a plant can also be influenced by the activity of its root or rhizosphere microbiota (Korenblum et al., 2020; Mangeot-Peter et al., 2020) and its direct abiotic environment (Bundy et al., 2009). For example, a study identified bacterial strains, isolated from *A. tinctoria* roots, which enhanced A/S contents in hairy-root cultures (Rat et al., 2021). Furthermore, in our previous study on the root metabolome and microbiome of *A. tinctoria* grown in greenhouse with different native soil microbiomes of diverse geographical origin, we identified the plant developmental stage as the prominent factor impacting the root metabolic composition, while the root microbiome was influenced by parameters such as the bulk soil microbiome as well as the developmental stage (Csorba et al., 2022). That study also revealed a correlation between specific microbiome taxa and metabolites of the A/S pathway indicating that certain taxa may stimulate A/S production or A/S metabolites might promote the proliferation of some specific microorganisms. In addition to abiotic environmental factors, biotic factors such as competition with other plant species (Leach et al., 2017), above- and below ground herbivory (Zhu et al., 2016; Hu et al., 2018) or attraction of pollinators (Borghi and Fernie, 2017) can shape the metabolic content and microbiome composition of plants.

**Figure 1:**
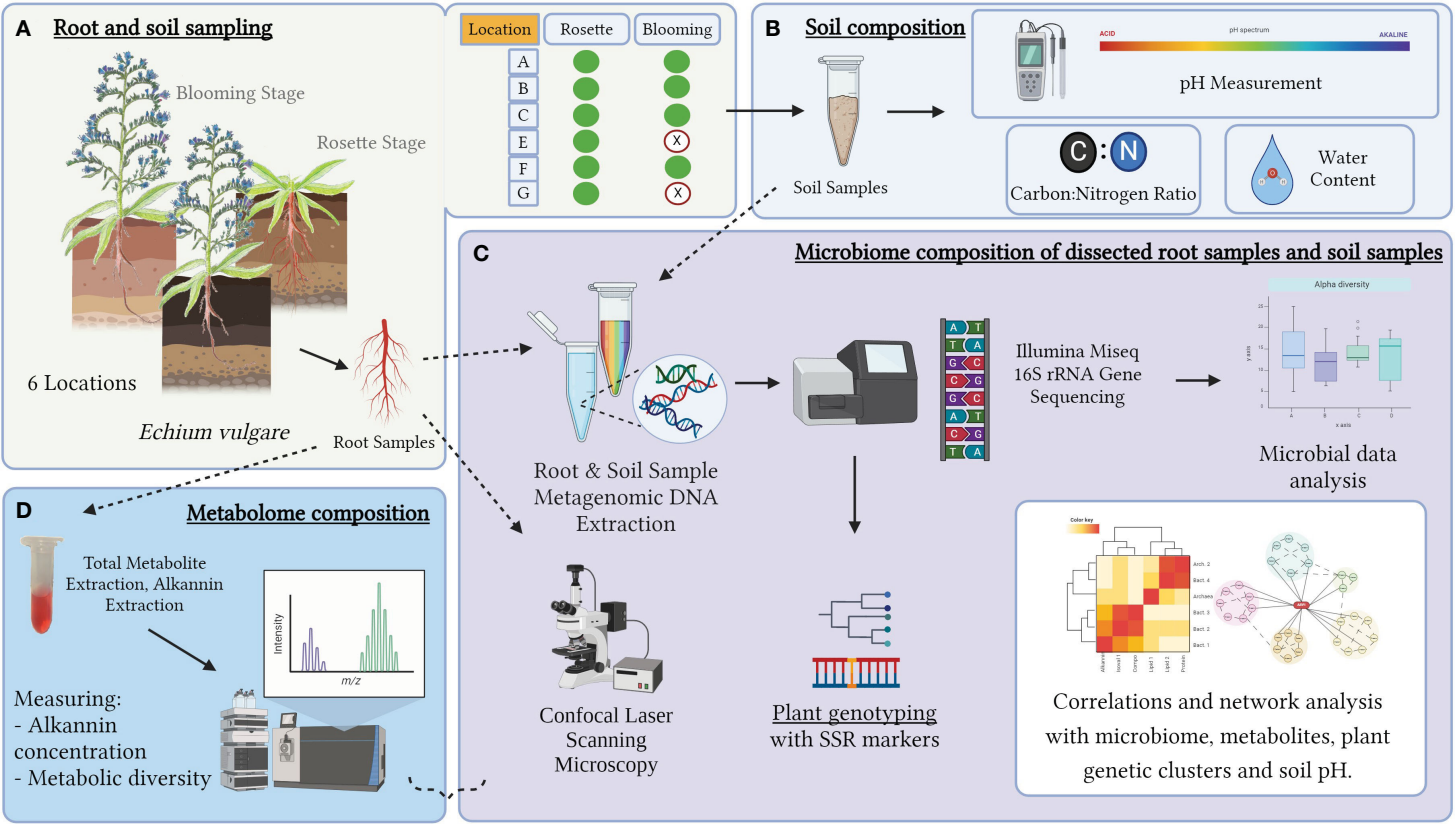
Graphical overview of the experiment, created with Biorender.com. **(A)** Experimental sampling setup, all variables of the analysis are depicted, the sample type of the roots and soils and the developmental stages of various geographical locations. **(B)** The soil composition measurements included in the experiment. **(C)** The research tools of microbiome composition analysis and the combined statistical analysis. **(D)** Short graphical description of the metabolome analysis pipeline.

In the present study, we formulated a hypothesis that native *E. vulgare* root and bulk soil samples collected from six geographical locations in Austria, each characterized by slightly different parameters, soil microbiomes, and soil biochemical compositions, would exhibit significant differences in their root-associated microbiome and metabolome. To elucidate potential microbial and metabolic changes associated with the developmental stages of *E. vulgare* roots, we sampled specimens from six stable populations both developmental stages (**Figure 1**). We aimed to investigate the potential influence of *E. vulgare* genetic variation between the populations, as well as the developmental stage within each population, on the microbial diversity, together with the primary and secondary metabolite content of the roots. Moreover, we anticipated discovering correlations and co-occurrences between root metabolites and microbial taxa in various stages and in plant genetic backgrounds. Our multidisciplinary approach can help to achieve enhanced comprehension of the intricate interplay among plant metabolism, environmental factors, and the associated microbiome.

## Materials and methods

### Sampling of *Echium vulgare* plants and bulk soil

Whole plants at two distinct growth stages (vegetative rosette and reproductive flowering stage), along with bulk soil corresponding to each root collected in each population from six different locations (locations A, B, C, E, F, G) in Austria in October and November 2017 (**Supplementary Figure 1**). The collection areas exhibited diverse habitat types (**Supplementary Table 1**). Among the selected *E. vulgare* populations, four consisted of plants both rosette and flowering stages, while the remaining areas provided only rosette stage plants. A total of 61 plants and the corresponding bulk soil material were sampled. Additional soil samples for chemical property analysis were collected from the same sites in April 2020.

### Root dissection and DNA extraction

**Figure 2:**
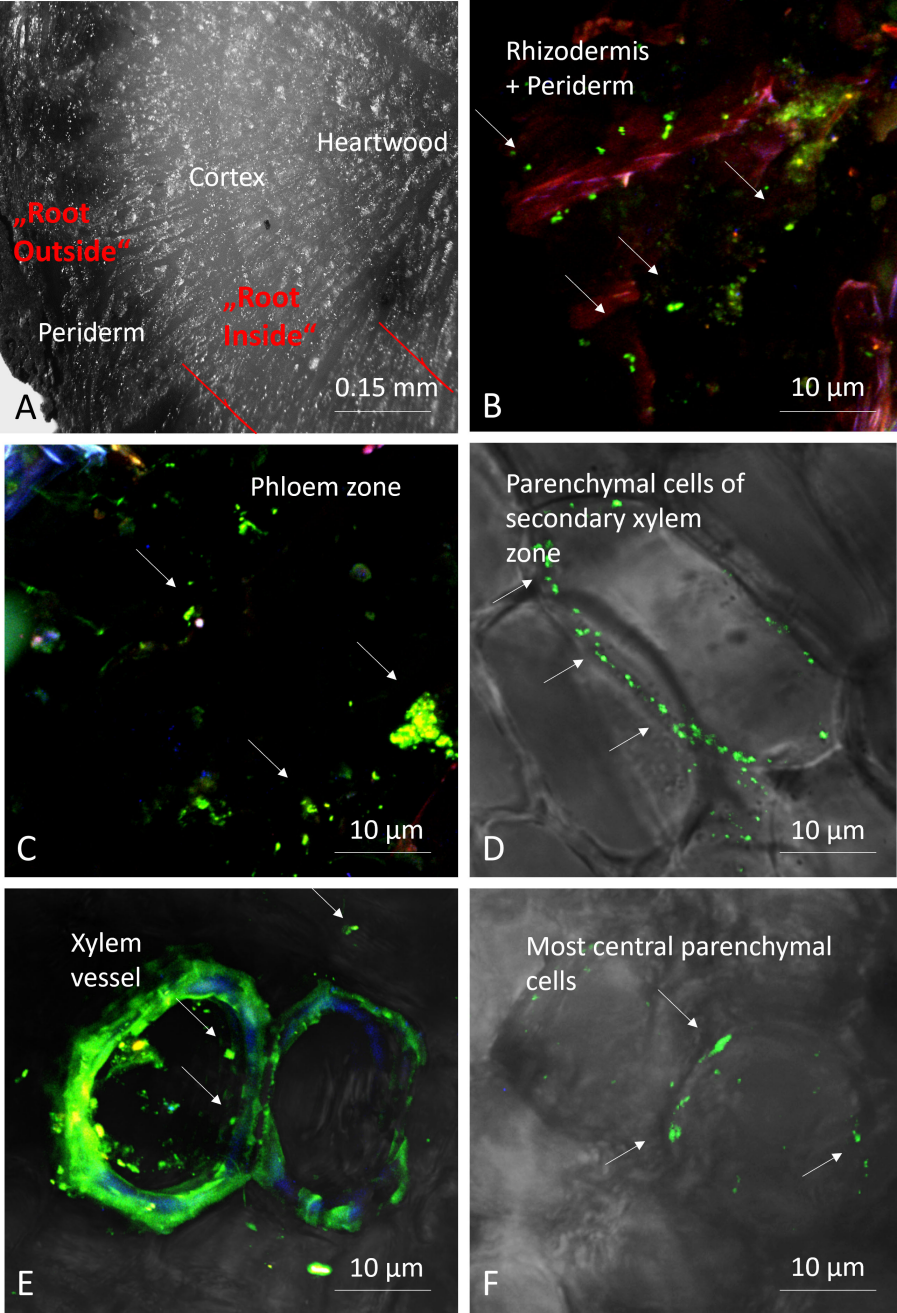
Microphotographs of root sections; transversal section of root showing the different zones using a stereomicroscope, with depicted red lines indicate site of dissection **(A)**, and confocal microscopy pictures after staining bacteria (green fluorescent) with Syto9^®^ **(B-F)**. Arrows: bacteria.

Plants were cut horizontally at the transition zone of the root, stem and leaves were discarded. Excess bulk soil attached to the roots was removed by manual shaking. Roots were placed in sterile 50 ml Falcon tubes and washed with sterile distilled water by placing on a tube roller mixer for 5 minutes. The surface structure and the hard-wooden property of the roots did not allow us to carry out sufficient surface sterilization without harming the periderm and its related microbiome. Therefore, we implemented a dissection step with sterile tools and defined two root sections to be handled separately. With the help of sterile scalpels, we separated the periderm including the secondary phloem, later referred to as “Root Outside – RO, from the secondary xylem or “Root Inside - RI” (**Figure 2A**), the hardened stele got discarded. Due to lack of surface sterilization the periderm samples contained sticky rhizoplane material. After dissection, the outer and inner tissues were handled separately and submitted instantly for fast freezing and DNA extraction. Soil total DNA was isolated by using FastDNA™ SPIN Kit for Soil and the FastPrep^®^ Instrument (MP Biomedicals, Santa Ana, CA, USA) by following manufacturer’s instructions. DNA was eluted from the Binding Matrix with 100 µl clinical-grade sterile distilled water (Aqua ad iniectabilia, Braun, Melsungen, Germany). The DNA from root samples was extracted by following a CTAB protocol (Csorba et al., 2022).

### Plant genotyping

PCR reactions on root DNA were performed by using FAM‐labelled M13 primers. The amplified products were separated on capillary sequencer and allelic configurations of each individual were determined using GeneMapper following the protocol already used on *A. tinctoria* by Ahmad et al. (2019). We genotyped 60 individuals of *E. vulgare* collected from six different locations in Austria using eight highly polymorphic DNA markers. Five of the Simple Sequence Repeat (SSR) markers (E3_40, E3_46, E3_56, E3_84, E3_91) were used from previous studies (Korbecka et al., 2003) while three of the markers (Col10, Col30b and Col9) were developed for other *Boraginaceae* species.

### Library preparation for 16S rRNA gene sequencing

Preparation of Illumina Miseq 16S rRNA amplicon gene V5-V7 region sequencing libraries contained all root and soil samples and were obtained through following a formerly used protocol by using the primer pair 799F-1175R (Hanshew et al., 2013; Csorba et al., 2022). As this primer pair is known to target also the plant-derived mitochondrial 18S sequences generating an ~800 bp product, separation and extraction of the ~400 bp 16S rRNA marker gene amplicon was needed with the help of agarose gel electrophoresis. In the second PCR step 48 different indexing tags were designed at the end of the 799F-1175R primer pair and individual amplicon libraries were planned accordingly. Pooled libraries were purified with Agencourt^®^ AMPure^®^ XP system and quantified with Qubit™ dsDNA HS Assay Kit on Qubit^®^ Fluorometer (ThermoFisher Scientific),. The libraries from all samples were submitted to LGC Genomics (Berlin, Germany) for Illumina MiSeq V3 (600 cycles) sequencing.

### Bioinformatic analysis

Raw reads were filtered with Bowtie2 v.2.3.4.3 (Langmead and Salzberg, 2012) to avoid the presence of Illumina’s PhiX contamination and quality was preliminarily checked with FastQC v.0.11.8. Primers were stripped using Cutadapt v.1.18 (Martin, 2011). Sequences were quality filtered, trimmed, denoised using default settings and amplicon sequence variants (ASVs) were generated with DADA2 v1.14 (Callahan et al., 2016). Denoised forward and reverse ASV sequences were merged, and chimeras were removed. Filtered ASVs were checked using Metaxa2 v2.2.1 (Bengtsson-Palme et al., 2016) for targeting the presence of V5-V7 16S rRNA regions in both archaeal and bacterial sequences. Taxonomic assignment of the 16S rRNA marker gene-based ASVs was performed using the RDP classifier (Wang et al., 2007) against the SILVA v138 (Quast et al., 2013) database. Bacterial ASV counts were imported into the R-4.0.3 statistical environment (R Core Team, 2021) for further analyses.

All statistical analyses concerning plant population genetics and the microbial community were conducted using R software packages (Version 3.5.1. and 4.0.3.) Contaminant sequences were filtered out using the decontam package (Davis et al., 2018), employing a prevalence method set at a threshold of 1%. Additionally, the RAM package (Chen et al., 2016) was utilized to exclude rare ASVs by implementing a maximum relative abundance threshold of 0.1%. Various functions of the package RAM were used for the visualization of taxon abundance, for calculating core taxa, and identifying indicator species. Relative abundances of the filtered table have been calculated by using the dplyr (Wickham et al., 2019) and phyloseq (McMurdie and Holmes, 2013) packages. Cumulative sum scaling before calculating beta diversity values was performed on the ASV table with the help of packages metagenomeseq (Paulson et al., 2013) and biomformat (McMurdie and Paulson, 2016). PERMANOVA on the resulting table was calculated by package vegan using the function adonis II. Normalization of the ASV table before alpha diversity analysis was performed through multiple rarefaction (permutation=999) with package rtk (Saary et al., 2017), by using the lowest number of reads. After conducting ANOVA, estimated marginal means were calculated in package emmeans (Lenth, 2023) as *post hoc* pairwise comparisons to confirm differences in means among all combinations of variables within the groups.

Indicator species were calculated with the multipatt function in indicspecies package (De Caceres and Legendre, 2009). This method utilizes permutation tests to assess the strength and statistical significance of associations between genus occurrence/abundance and groups of sites. In the group.indicators RAM/multipatt analysis, thresholds were set at 0.85 for indicator specificity, 0.8 for indicator fidelity, 0.8 for the association strength of the combined specificity and fidelity, and a significance level of 0.05 for p-values. The calculation of reproducibly occurring ASVs (rASVs) was conducted on DeSeq2 (Love et al., 2014) normalized microbiome data. This was achieved by generating a hybrid artificial factor, which combined the factors of soil pH, sample types, and locations using the R package dplyr. Subsequently, the core.OTU function from RAM was employed, setting a threshold of 4/6. This threshold determined the ASVs present in 4 out of 6 replicates within each artificial factor, and 0.05 at p values (Pfeiffer et al., 2017; Csorba et al., 2022). The significantly differentially abundant rASVs were calculated using manyglm analysis (multivariate glm in the mvabund package), considering both factors. Subsequently, an ANOVA based on manyglm with 999 permutations was conducted. Shared rASVs and core microbiome were analyzed through Venn diagrams with venny 2.1.0.

The bacterial root microbial rASVs and UHPLC-HRMS metabolites were correlated, and strongly correlating (r>|0.7|) pairs were used to test for co-occuring factors. Co-occurrence networks were established based on correlation done in the R package psych (Revelle and Revelle, 2015), random forests were trained and built with R packages parallel, caret (Kuhn, 2008), tidyR (Wickham et al., 2023) and rfPermute. Networks were calculated, analyzed and visualized by using igraph (Csárdi et. al., 2006) and tidygraph (Pedersen, 2023) packages.

### Stereomicroscopy and confocal laser scanning microscopy

To locate and visualize microorganisms in the different parts of the root tissues, we scanned cross-sections obtained from freshly collected roots from location C. These sections were observed using a stereomicroscope (Olympus SZX16) and a confocal laser scanning microscope (Olympus Fluoview FV1000 with multiline laser FV5-LAMAR-2 HeNe(G) and laser FV10-LAHEG230–2) after staining with 3.34 µM Syto9^®^ (ThermoFisher) in PBS, enabling visualization of microorganisms residing inside the root. For confocal microcopy X, Y, Z pictures were taken at 405, 488, 594 nm and with different objectives and then merged (RGB) and observed using Imaris software (Oxford Instruments). Pictures were cropped, whole pictures were sharpened, and the light/contrast balance was improved on whole pictures.

### Soil sampling and chemical property analysis

Soil samples were collected using a 1 cm diameter soil corer from the sampling sites. Within a 1x1 m square area nine individual points from the top soil layer of 10–15 cm in depth were taken and then pooled by location. Subsequently, the soil samples were sifted through a 2 mm sieve, and the resulting finer fractions were homogenized using a ball mixer (Retsch mill) for further measurements. The pH of the samples was measured by following the protocol of ÖNORM L 1083. The pH classification is based on the recommendation of the United States Department of Agriculture (Soil Science Division Staff, 2017). Gravimetric soil water content (GWC) was determined by oven drying 20 g of air-dried samples at 105°C for 48 h. Carbonate content of the samples was measured by the Scheibler method (ÖNORM L 1084). Total carbon and total nitrogen of the air-dried and at 105°C pre-dried soils were measured by dry combustion. The C:N ratio was calculated by dividing the soil organic carbon content (SOC) and the soil nitrogen content (%N).

### Chemicals and sample preparation for metabolite analysis

The A/S standards, chemicals and protocols for metabolite extraction used were the same as previously described in former studies (Tappeiner et al., 2014; Csorba et al., 2022). For NMR spectroscopy analysis, 600 µL of the crude extract, prepared as described in our previous *Alkanna tinctoria* study (Csorba et al., 2022), were evaporated under a stream of nitrogen and reconstituted in methanol-d_4_ containing 0.001% TSP.

### Ultra-high performance liquid chromatography - high resolution mass spectrometry method

The UHPLC-HRMS method used in this study, including the acquisition parameters and subsequent data extraction, was previously reported (Csorba et al., 2022). In short, the UHPLC-HRMS data of the *E. vulgare* root extracts were recorded on an LTQ Orbitrap Discovery (Thermo Scientific, Waltham, Massachusetts, USA) instrument with the chromatographic separation being performed by an Acquity UPLC HSS C18 SB 1.8 μm 2.1 x 100 mm (Waters, Milford, Massachusetts, USA) column. Solvents used were ultrapure water (A) and methanol (B), with 0.1% formic acid added in both cases. The gradient elution program was the following: 0 min 95A/5B, 1 min 50A/50B, 8 min 0A/100B, 13 min 0A/100B, 13.01 min 95A/5B, 16 min 95A/5B. The six most intense ions in each full scan (m/z 80–1000) were subjected to MS/MS. Data processing was performed using Xcalibur (Thermo Scientific, USA) and the XCMS Online platform (The Scripps Research Institute, USA).

### Metabolite annotation and statistical analysis of UHPLC-HRMS-derived metabolomics data

UHPLC-HRMS data alignment and feature extraction were performed utilizing the XCMS Online platform (The Scripps Research Institute, USA). Features were detected via the centWave algorithm (Tautenhahn et al., 2008) based on m/z values and their respective retention times. The maximum tolerated m/z deviation was set to 2.5 ppm in consecutive scans, with the signal to noise threshold set to 10. Only chromatographic peaks with a width of at least 5 s were extracted. Obiwarp (Prince and Marcotte, 2006) was the method used for retention time alignment, 5 s being the maximum allowed retention time shift. Following the feature extraction, the UHPLC-HRMS data were passed through a relative standard deviation in the QC filter of 20%. Herein a feature is defined as a unique combination of m/z ratio and retention time; a single metabolite can yield multiple features depending on its structure and analysis conditions. Each feature with a higher relative standard deviation was removed from the dataset. The data were normalized by median and subjected to log transformation, which resulted in a normally distributed data matrix. One-way ANOVA, fold change analyses and (s)PLS-DA on different subsets of the data, as well as correlation analyses based on the Pearson correlation coefficient were done with the help of MetaboAnalyst 5.0 (Pang et al., 2021b) using its single-factor and multi-factor modules. Compound annotation was done by matching m/z values and MS/MS fragmentation spectra to those found in literature, the mzCloud database connected to Compound Discoverer 3.3 (Thermo Fisher Scientific, Waltham, Massachusetts, USA), MassBank, MoNA or via automatic MS/MS matching through the GNPS platform (Nothias et al., 2020), while also taking into account retention times. GNPS was used to generate molecular networks which were then further developed in Cytoscape 3.9.1 (Shannon et al., 2003).

### HPLC-UV/Vis method for quantitation of A/S, chiral analysis and NMR analysis

In order to quantify the contents of A/S we used the method described in our previous study (Csorba et al., 2022) and an adapted (Tappeiner et al., 2014).

NMR spectra were recorded on a Varian/Agilent (Palo Alto, California, USA) 600 MHz instrument utilizing the OneNMR probe. The pulse sequence chosen for recording the spectra was a PRESAT sequence with pre-saturation pulses for suppressing solvent resonances. The number of scans was set to 256, with the relaxation delay of 2 s and the spectral width of 9615.4 Hz. Acquisition took 1.7 s per scan and resulted in spectra that were linearly predicted to 64k data points. All spectra were acquired in a temperature-controlled environment, at 298.15 K. A delay of 10 min was allowed for the sample to reach thermal equilibrium, which was followed by shimming of the magnet and spectral acquisition. Processing of the spectra was performed by a combination of Agilent VnmrJ 4.2 and MestReNova 14.2.1 (Mestrelab Research, S.L., Santiago de Compostela, Spain) software.

### Data availability

The microbiome sequencing data were deposited in NCBI SRA and are available under the BioProject accession number PRJNA1010776. The metabolomics dataset is available at the NIH Common Fund’s National Metabolomics Data Repository (NMDR) website, the Metabolomics Workbench, https://www.metabolomicsworkbench.org, where it has been assigned Project ID PR001283. The data can be accessed directly via its Project DOI: 10.21228/M8VX1B. The codes of microbiome and co-occurrence network analysis are available in Zenodo under DOI: 10.5281/zenodo.11281718.

## Results

### Soil composition differed across geographical locations

According to the measured soil pH, the six locations were grouped in three soil types, neutral (location A, B and C), slightly alkaline (location E and F) and moderately acidic (location G). The nitrogen content, the SOC, the carbon-nitrogen ratio, and the GWC across the six locations based on Kruskal-Wallis test were significantly not different (p=0.415) (**Table 1**). However, SOC was significantly different between the three soil pH categories (ANOVA, p =0.028).

**Table 1:** Soil composition factors from the different locations.

Location	pH	Denomination - pH	%N	SOC	C:N ratio	GWC (%)
**A**	7.318	Neutral	0.18	3.09	18:1	3.1
**B**	7.339	Neutral	0.13	1.89	16:1	1.42
**C**	7.372	Neutral	0.16	2.51	16:1	2.88
**E**	7.488	Slightly alkaline	0.32	5.27	17:1	4.16
**F**	7.486	Slightly alkaline	0.24	5.07	21:1	2.04
**G**	5.903	Moderately acidic	0.16	3.07	19:1	1.42

### Two genetic clusters were identified through plant genotyping

Genetic differentiation estimates grouped the 60 collected individuals in two genetic clusters (Cluster I: n = 24, Cluster II: n = 36) (**Supplementary Figure 2A**). In addition, population differentiation estimates based on the pairwise comparison (Fst) showed the same two-group genetic distinction. Fst values were lower between populations from the same genetic cluster, and indicated, that populations A and B are genetically closer to each other in Cluster I, than C, E and F in Cluster II (**Supplementary Figure 2B**).

### Confocal scanning laser fluorescence microscopy verified the presence of endophytes

To confirm the presence of endophytes in the roots of *E. vulgare*, we performed stereomicroscopy and confocal scanning laser fluorescent microscopy on the top sections of some root samples. The stained regions showed green, fluorescent cells in the intracellular space of the root tissues, which confirmed the presence of microorganisms inside the separated and respective root tissues of *E. vulgare* (**Figure 2**).

### Microbial diversity differed between soil and root samples and were influenced by location, soil pH and plant genetic clusters

**Figure 3:**
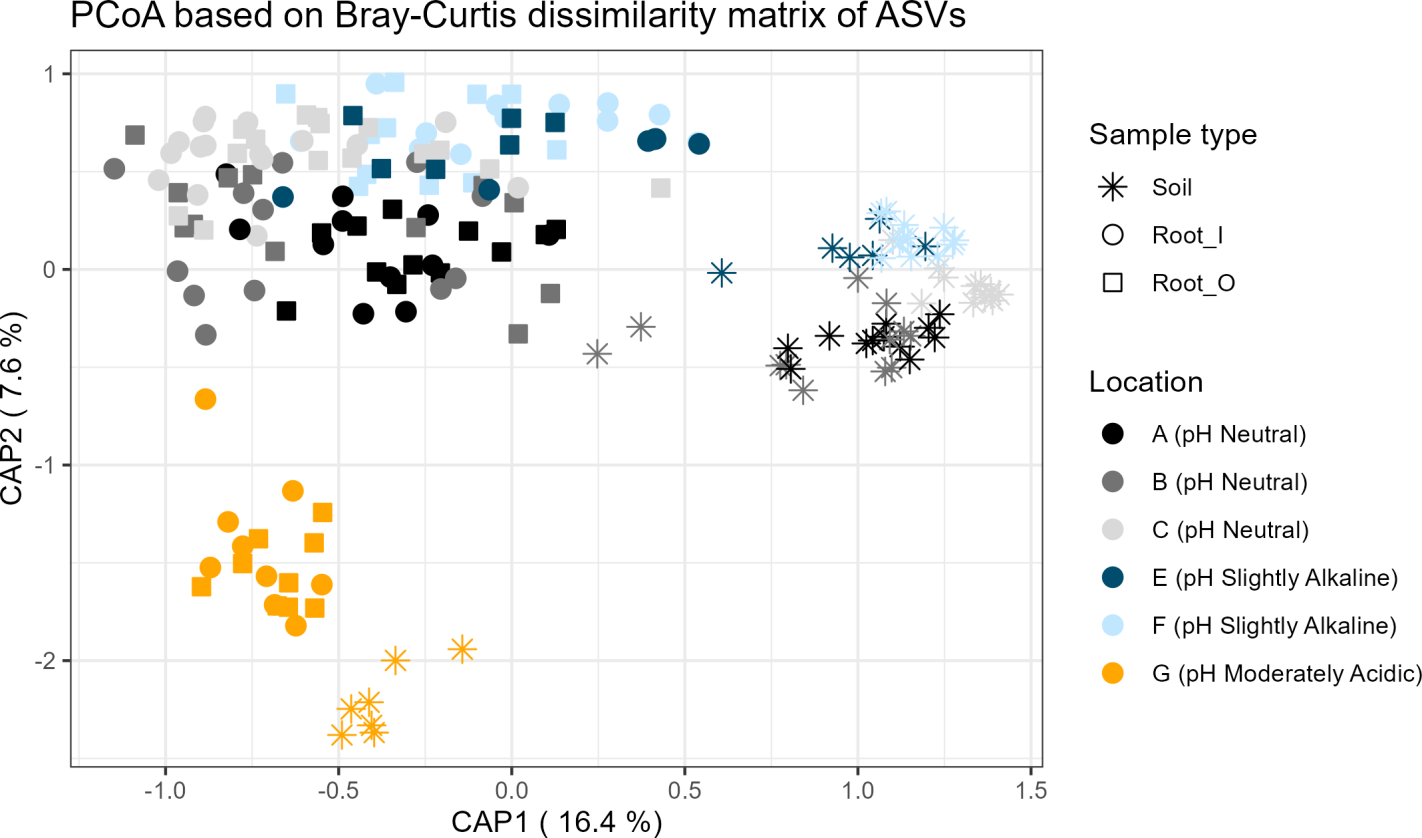
Beta diversity principal component analysis based on Bray-Curtis distances. The first principal component (CAP1) is explaining 16.4% of the variance. The grey-scale colors are the locations with soils with neutral pH, blue colored locations had slightly acidic pH and the yellow color represents the moderately acidic soil conditions. The star symbols represent bulk soil samples, filled circles are Root O (outer section of the root) and filled rectangles are Root I (inner root sections).

As a result of sequencing the V5-V7 region of the 16S rRNA marker gene, from 44680 total ASVs after decontamination and filtering, 7737 bacterial and archaeal ASVs were identified in 193 samples. Based on regression analysis including all samples, the sample type (root and bulk soil) was the most important factor influencing the Simpson´s diversity index and microbial richness, while developmental stages were non-significant in the alpha diversity measures (**Supplementary Table 3**). Regarding the microbiome of the inner root (RI) samples, the soil pH was the main factor on the alpha diversity measures; while in the outer root (RO) and soil samples, the geographical location was the most important significant factor (**Supplementary Table 3**, **Supplementary Figure 3**). According to PERMANOVA based on Bray-Curtis distances on the cumulative sum scaling normalized microbial data, the microbiome of the sample types (root/soil), RI, RO and soil, were significantly different (**Table 2**). However, *post hoc* pairwise comparisons revealed that bulk soil samples had dissimilar beta diversity to all root samples, while RI and RO samples hosted similar microbiomes (**Supplementary Table 2**). Furthermore, prokaryotic beta-diversity was significantly influenced by location, soil pH and the location groups corresponding to the genetic clusters in all sample types, while the developmental stage did not significantly influence the microbiome (**Table 2**). In the pairwise analysis of Bray-Curtis dissimilarity values, it was determined that the microbiome samples of roots and soil from location G (slightly acidic) were distinct from those of other locations (**Supplementary Table 2**, **Figure 3**).

**Table 2:** PERMANOVA (adonis2) on normalized data based on Bray distances.

	Variable	R^2^	F	P
**Bacteria, All samples**	Sample type	0.046	4.993	0.001***
**Bacteria, Root inside**	Location	0.281	4.060	<0.0001***
	Soil pH	0.017	5.749	<0.0001***
	Genetic cluster	0.039	2.642	<0.0001***
	Developmental stage	0.031	1.364	0.087
**Bacteria, Root outside**	Location	0.305	4.565	<0.0001***
	Soil pH	0.169	5.889	<0.0001***
	Genetic cluster	0.053	3.673	<0.0001***
	Developmental stage	0.022	0.980	0.449
**Bacteria, Soil**	Location	0.195	2.688	0.001***
	Soil pH	0.125	4.204	0.001***
	Genetic cluster	0.028	1.822	0.024*
	Developmental stage	0.405	0.021	0.081

a *p<0.05, ***p<0.001.

Developmental stages were analysed in a subset of the locations containing both stages^a^.

### Taxonomic composition analyses highlighted the effects of environmental factors on soil and root microbiome while specific indicator taxa are identified based on soil pH

We determined the prokaryotic microbial composition at the genus level of bulk soil, RI and RO samples. The RO samples also had unremovable rhizoplane particles attached to the periderm surface after dissection. In total, we assigned our filtered data to 25 prokaryotic phyla, 59 classes, 109 orders, 160 families, 333 genera and 7737 ASVs. The RI and RO samples contained primarily Proteobacteria (Pseudomonadota according to the valid phylum names (Oren and Garrity, 2021)), Actinobacteriota (Actinomycetota) and Firmicutes (Bacillota), while the soil samples were dominated by Actinobacteriota (Actinomycetota), Proteobacteria (Pseudomonadota) and Bacteroidota. We only detected one archaeal phylum, Crenarchaeota (Thermoproteota), which had relative abundances higher than 5% in all locations with neutral and alkaline soils (locations A, B, C, E, F), while in the acidic soil (location G) it was only 2%. The taxonomic composition at genus level consisted of highly varying relative abundances in the individual sample groups, we visualized them according to locations and developmental stages (**Supplementary Figure 4**) and according to plant genetic clusters (**Supplementary Figure 5**). The amount of ASVs taxonomically not assigned (NA) to any genera was relatively high (in total 29% of the whole relative abundance), especially in soil samples (all > 25% relative abundance, some >50%), however these were all assigned at higher taxonomical ranks to known prokaryotic taxa.

**Figure 4:**
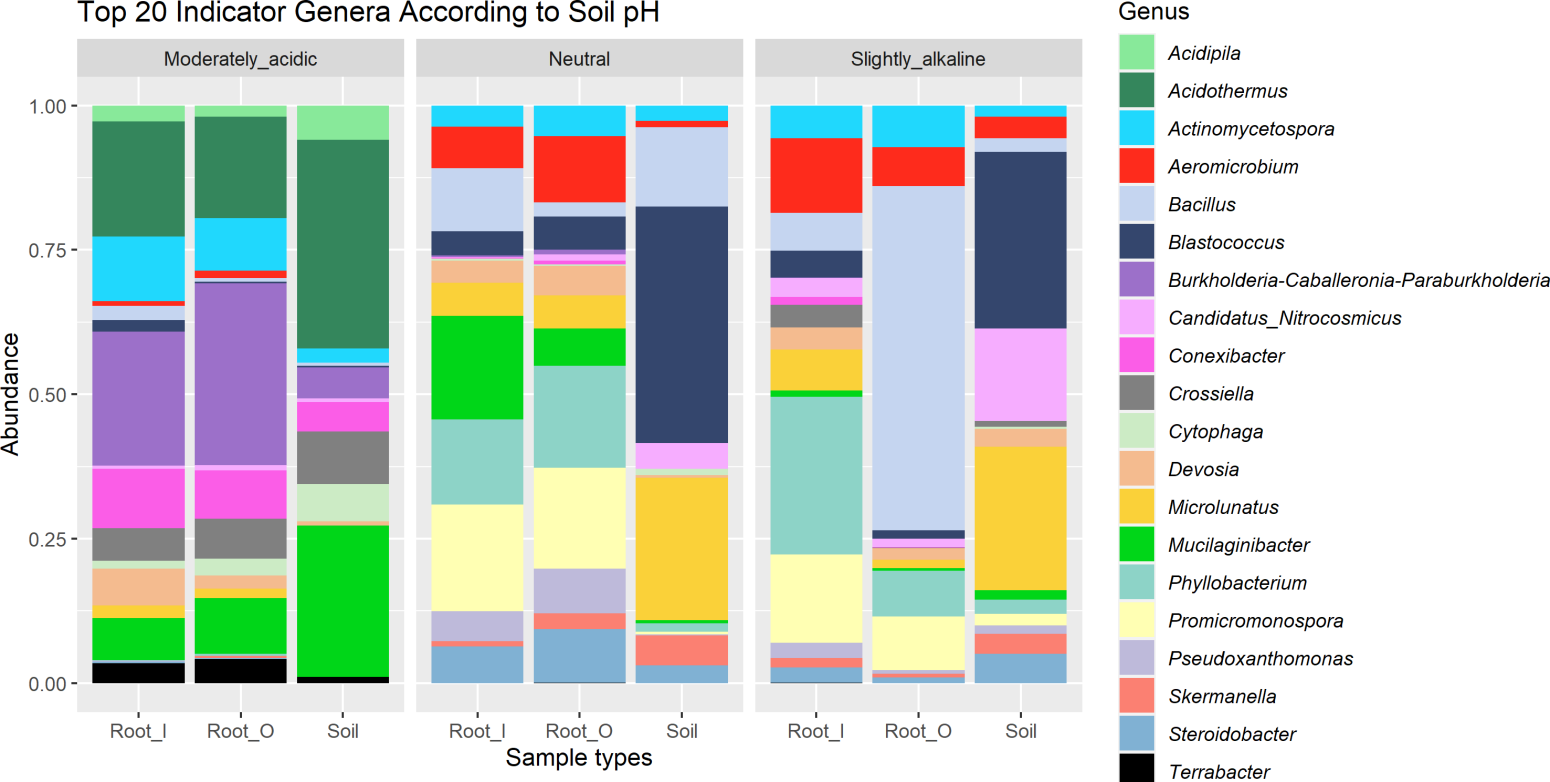
Relative abundance of top 20 indicator genera in different soil pH categories. Moderately acidic soil was found in location G, neutral in location **(A, B)** and **(C)**, while location **(E)** and **(F)** had soil with slightly alkaline properties.

By using the soil pH as an indicator factor in our indicator taxa analysis, with thresholds of 0.8 association strength of the combination of fidelity and specificity on rarefacted data, we identified genera which were only found in a certain soil pH range (**Figure 4**, **Table 3**). In roots of plants sampled in the moderately acidic environment (G) and in the corresponding bulk soil samples, *Acidothermus*, *Acidipila* and *Terrabacter* genera were exclusively found. The root samples from the acidic environment (G) also showed a higher abundance of *Burkholderia-Caballeronia-Paraburkholderia* than in other soils. Indicator taxa of both root and soil samples with high relative abundance from non-acidic (neutral and slightly alkaline) soils (A, B, C, E, F) were *Bacillus*, *Aeromicrobium*, *Devosia*, *Blastococcus* and *Microlunatus*. The taxa *Blastococcus* and *Microlunatus* were consistently found in bulk soil samples from all locations, except in location G, which had acidic properties. Root samples collected from non-acidic (neutral and slightly alkaline) soils (A, B, C, E, F) contained the indicator genera *Phyllobacterium*, *Promicromonospora*, *Pseudoxanthomonas* and *Skermanella*. When using the same threshold of association strength in analysis on soil pH indicator species, *Actinomycetospora iriomotensis* and *Mycobacterium paraterrae* were indicator species of root and soil samples of the moderately acidic environment (G). By using the same thresholds in the indicator taxa calculation, geographical locations, developmental stages and genetic clusters did not result in identified indicator taxa at any taxonomic level.

**Table 3:** Association strength values of top 20 indicator taxa of soil pH, visualized on **Figure 4**.

Genus name	Specificity	Fidelity	Association strength	P value
***Acidothermus***	1	0.96	0.98	0.001
***Acidipila***	1	0.88	0.938	0.001
***Burkholderia-Caballeronia-Paraburkholderia***	0.9307	0.92	0.925	0.001
***Cytophaga***	0.8831	0.92	0.901	0.001
***Phyllobacterium***	0.9978	0.9281	0.962	0.001
***Microlunatus***	0.9742	0.8802	0.926	0.001
***Steroidobacter***	0.9878	0.8144	0.897	0.001
***Mucilaginibacter***	0.7772	1	0.882	0.001
***Blastococcus***	0.9795	0.7844	0.877	0.001
***Aeromicrobium***	0.9769	0.7844	0.875	0.001
***Terrabacter***	0.9862	0.76	0.866	0.001
***Devosia***	0.8202	0.8974	0.858	0.001
***Actinomycetospora***	0.848	0.833	0.841	0.001
***Promicromonospora***	1	0.7006	0.837	0.001
***Bacillus***	0.8196	0.8491	0.834	0.003
***Pseudoxanthomonas***	0.9954	0.6946	0.832	0.001
***Cand.Nitrosocosmicus***	0.7675	0.8868	0.825	0.001
***Crossiella***	0.7655	0.88	0.821	0.001
***Conexibacter***	0.8979	0.72	0.804	0.001
***Skermanella***	0.9891	0.6527	0.803	0.001

### Transient and core microbiome ASVs revealed shared taxa between the samples of different variables

**Figure 5:**
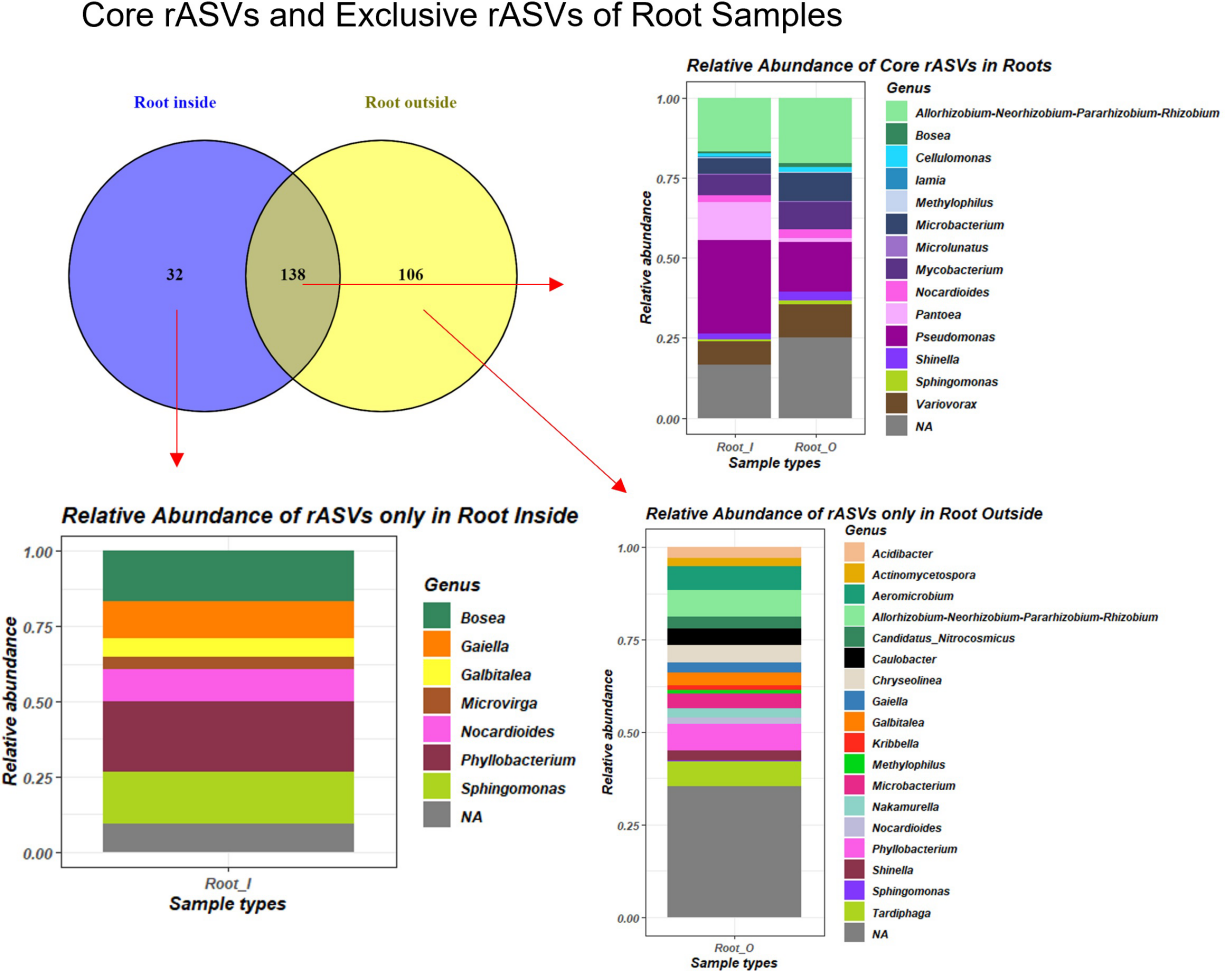
Venn diagram of core microbiome of *E. vulgare* roots independently of factors, based on shared reproducibly occurring ASVs (rASVs) and their relative abundance barplots.

We calculated reproducibly occurring ASVs (rASVs), which were present in at least four out of six replicates in each group. We analyzed the DESeq2 normalized rASV tables and identified the core plant rASVs, which consisted of 138 ASVs present in all root samples independently of their origin. In our results we applied the definition of complete core microbiota as a constant microbial community associated with the plant host independently of all other factors or in the soil specific to the local environmental conditions (Berg et al., 2020). The root-associated core rASVs belonged to genera *Allorhizobium-Neorhizobium-Pararhizobium-Rhizobium*, *Pseudomonas*, *Microbacterium, Mycobacterium* and *Variovorax* in the highest relative abundance among other lower abundance and unassigned genera (**Figure 5**). However, the RO samples also contained rhizoplane particles, therefore the 32 rASVs from the RI were the only taxa purely from root tissues. These 32 rASVs were from the genera *Bosea*, *Gaiella*, *Galbitalea*, *Microvirga*, *Nocardioides*, *Phyllobacterium* and *Sphingomonas*. Furthermore, 50 rASVs were shared between soil and root samples in soil pH subsets of the data (**Supplementary Figure 6**), these rASVs were assigned to seven genera (*Allorhizobium-Neorhizobium-Pararhizobium-Rhizobium*, *Cellulomonas*, *Microbacterium*, *Mycobacterium*, *Nocardioides*, *Pseudomonas* and *Variovorax*). Transient microbiota per definition was calculated as a specialized section of microbiota changing over time or between environmental conditions (Berg et al., 2020). We determined transient microbiota consisting of rASVs exclusive to specific soil pH conditions (**Supplementary Figure 7**). The 280 rASVs exclusively present in root and soil samples from the moderately acidic soil (location G, **Supplementary Figure 6**) were assigned to 50 genera (**Supplementary Figure 7**). Some of these genera, such as *Acidipila*, *Acidothermus*, *Burkholderia-Caballeronia-Paraburkholderia*, *Conexibacter*, *Crossiella*, *Cytophaga* and *Terrabacter* were all found to be exclusively present under acidic soil conditions both in root and soil samples based on their unshared rASVs and on the indicator genera analysis as well. Similarly, root and soil samples from slightly alkaline soil conditions (G) harbored 239 exclusive rASVs (**Supplementary Figure 6**) in their transient microbiota which belonged to 37 genera (**Supplementary Figure 7**). The genera *Aeromicrobium* and *Bacillus* were both part of the alkaline-soil-associated transient microbiota as well as indicator taxa.

### The root metabolome of *E. vulgare* was significantly influenced by plant developmental stage, location and soil pH

Out of all the features extracted from the UHPLC-HRMS raw data, a total of 1455 features got through our 20% relative standard deviation in the QC filter. According to PERMANOVA based on Euclidean distances on the log-normalized and center-scaled LC-MS data, location, soil pH and developmental stage significantly influenced the root metabolomic diversity, while samples from the two genetic clusters had similar root metabolome compositions (**Table 4**). Correlation analyses based on the Pearson r coefficient revealed 276 UHPLC-HRMS features strongly correlated (|r|≥0.7) with the developmental stage. For the remaining factors, no features exhibited strong correlations. However, 10 features showed correlation (0.6<|r|<0.7) with location, and 4 features with soil pH. For each of the factors, we ranked the features associated with them using random forest mean decrease accuracy and annotated some of the top hits. In the case of developmental stage, random forest highlighted the genus-characteristic alkaloid echimidine (r=0.89) and a hexosamine (r=0.78), which could be either glucosamine, galactosamine or mannosamine, as these are indistinguishable by the analytical method used. Another alkaloid, viridiflorine (r=0.21) was found to be associated with soil pH, as were 2-aminobutyric acid (r=0.13) and proline (r=0.07). Anthranilic acid (r=-0.61) and the tentatively annotated methyl dihydrojasmonate (r=-0.63) appeared as metabolites most influenced by the location factor.

**Table 4:** PERMANOVA (adonis2) on normalized UHPLC-HRMS metabolome data based on Euclidean distances.

	Variable	R^2^	F	P
Metabolites, Root total	Location	0.223	2.987	<0.0001***
	Soil pH	0.157	5.138	0.0001***
	Genetic cluster	0.018	1.198	0.205
	Developmental stage	0.114	5.555	<0.0001***

***p<0.001.

**Figure 6:**
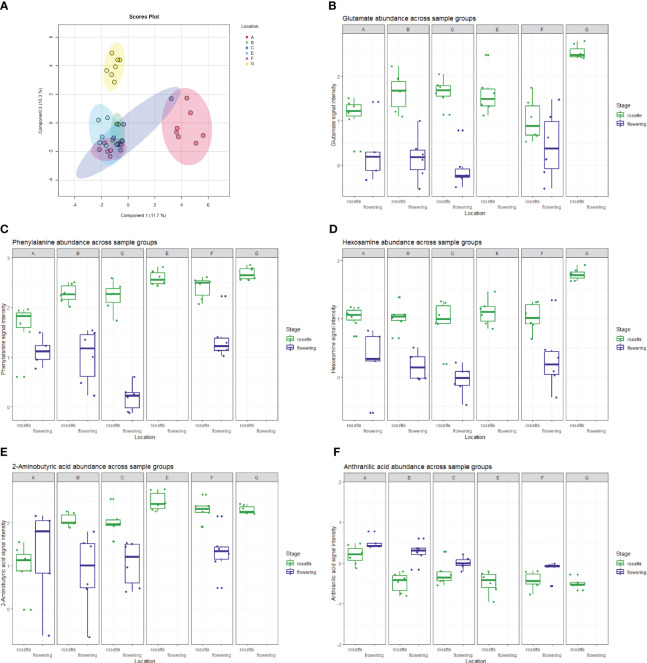
**(A)** The (s)PLS-DA plot showing the separation of *E. vulgare* rosette samples in the 6 locations studied. Location G, the only location with moderately acidic pH, is clearly separated from all the other locations. A selection of metabolites driving separation between sample groups, comprising of **(B)** glutamate, **(C)** phenylalanine, **(D)** hexosamine, **(E)** 2-aminobutyric acid and **(F)** anthranilic acid is shown through boxplots denoting their abundance across sample groups.

In a data subset that contained only root samples of flowering plants, one-way ANOVA analysis of samples from different locations highlighted 11 statistically significant (p<0.05) features, among which were the already mentioned anthranilic acid and methyl dihydrojasmonate. In contrast, when only root samples of rosette-staged plants were included, the same type of analysis revealed 737 statistically significant features. Topping the list of significant features with the lowest p-value in the entire dataset was the protonated form of phenylalanine (p<<0.01), while the Fisher *post-hoc* tests for the feature revealed that the mean signal intensities for this metabolite between the two developmental stages vary in a statistically significant way across all the locations. Within the rosette developmental stage, phenylalanine abundance was statistically significant (p<<0.01) when samples from different locations were compared. A metastable ion at 120 m/z, which arose from phenylalanine fragmentation, was also observed and was one of the top features in the above-mentioned one-way ANOVA analyses. The abundance of phenylalanine, alanine, glutamate, leucine, threonine and valine, but also the dipeptides alanylproline and valylproline was highest in location G and was found significant in one-way ANOVA analysis of rosette-staged plant roots compared to other locations (p<0.01 in all cases). Proline was an exception; it was found significant but was not found in highest quantities in roots from the mentioned location. In the correlation analysis based on the Pearson r coefficient, glutamic acid had the highest positive correlation (r=0.67) with the location factor out of all features in the dataset. The listed amino acids were among the metabolic features responsible for the separation of location G in our rosette stage sparse PLS-DA plot (**Figure 6**). Component 1 explained 11.7% of the variance and some of the variables with the highest loadings within said component included 2-aminobutyric acid, phenylalanine, arachidonic acid, anthranillic acid and glutamate. The 2-aminobutyric acid was significantly lower in concentration in location A, anthranillic acid reached a significantly increased level in location A compared to other locations, while phenylalanine and glutamate acid were most abundant in location G compared to other locations (**Figure 6**).

### Pyrrolizidine alkaloid analyses revealed a correlation to plant developmental stage

**Figure 7:**
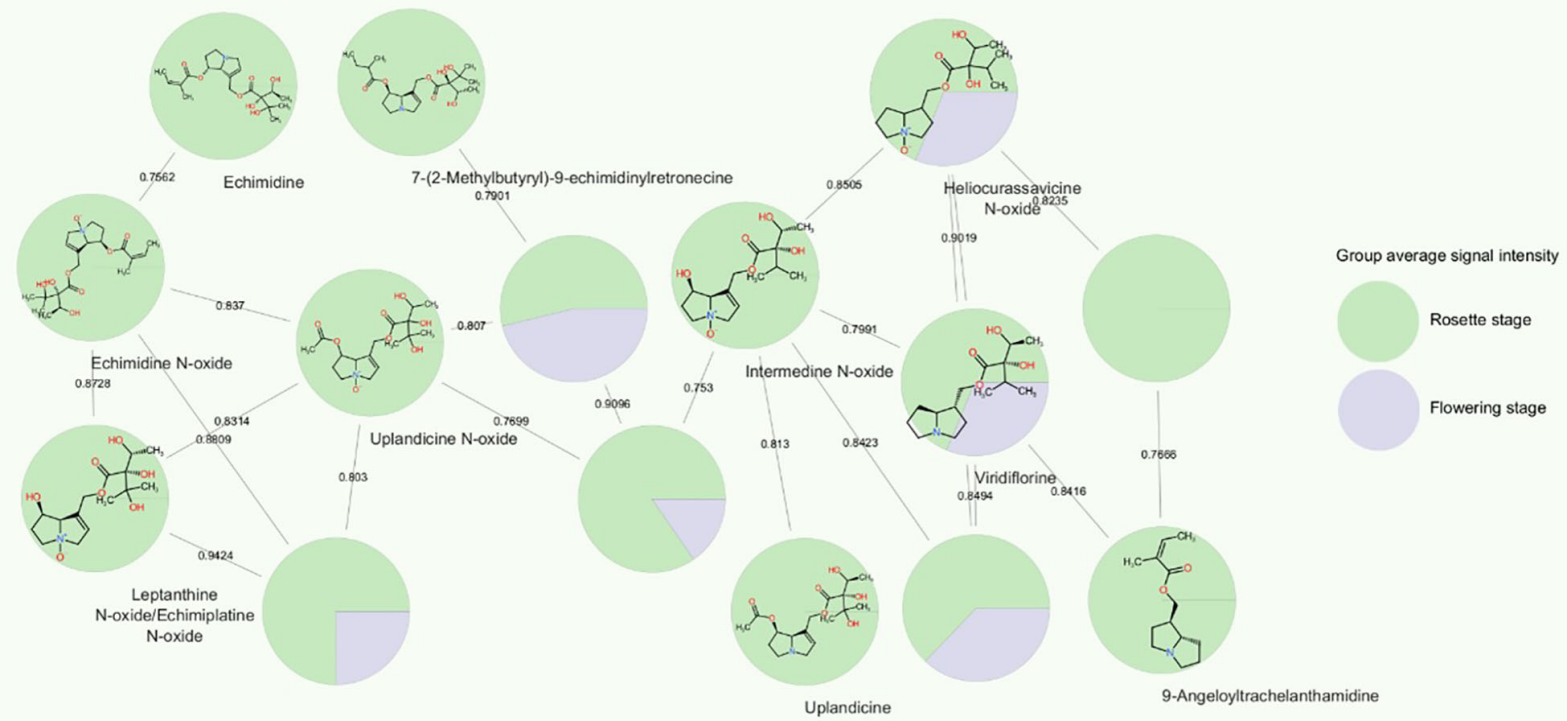
Cytoscape molecular subnetwork of pyrrolizidine alkaloids and their signal intensities in *E. vulgare* plant roots collected from location C. The network was created based on cosine scores that represented the similarity of MS/MS fragmentation spectra of compounds, i.e. their structural similarity. The nodes are shown as pie charts that reflect the ratio of average signal abundance in rosette *E. vulgare* roots and roots of flowering plants.

The PA echimidine exhibited one of the strongest correlations with the developmental stage, with its protonated ion ranking third among the 1455 features included in the correlation analysis. We annotated several PAs based on their MS/MS fragmentation spectra, as well as their precursors arginine and the tentatively annotated ornithine. All of these metabolites were observed to exhibit a positive correlation with developmental stage, being more abundant in the rosette stage root extracts, with fold changes FC ≤ 0.5 and p<0.05 in t-tests (**Supplementary Table 4**). A subnetwork, obtained through GNPS/Cytoscape molecular networking based on MS/MS fragmentation similarity (**Figure 7**), immediately highlighted the increased abundance of all annotated PAs in the *E. vulgare* rosette-staged root samples compared to roots from the flowering stage. The positive correlations between PAs and the developmental stage were very weak in the case of leptanthine N-oxide/echimiplatine N-oxide (r=0.20), weak for uplandicine N-oxide (r=0.32), intermedine N-oxide (r=0.41) and echimidine N-oxide (r=0.46), moderate for heliocurassavicine N-oxide (r=0.57), acetylechimidine N-oxide (r=0.58) viridiflorine (r=0.59), 9-angeloyltrachelanthamidine (r=0.63), 7-(2-methylbutyryl)-9-echimidinylretronecine (r=0.66) and uplandicine (r=0.67), and high for echiuplatine (r=0.73), acetylechimidine (r=0.76) and the already mentioned echimidine. We also tentatively annotated 7-(2-methylbutyryl)-9-echimidinylretronecine N-oxide without having access to its MS/MS spectrum. This alkaloid was annotated based on the exact mass of its protonated ion, the similarity of its MS/MS spectrum to that of echimidine N-oxide (and the fact that the compound eluted together with 7-(2-methylbutyryl)-9-echimidinylretronecine. The N-oxide was also positively correlated with the developmental stage (r=0.40) and was present in higher amount in the rosette-staged root samples. Intermedine/lycopsamine and leptanthine were also found in the root samples. The full list of compounds annotated is available in **Supplementary Table 5**.

In addition to being influenced by the developmental stage, all the PAs mentioned, except echimidine, 7-(2-methylbutyryl)-9-echimidinylretronecine and intermedine-N-oxide, were also found to be statistically significant in the one-way ANOVA analysis when considering only rosette-staged root extracts. The location also influenced their abundance, but not in the same way for all the compounds. For example, acetylechimidine was present in similar amounts in samples from locations A, C, E, and F, less so in G, and its signal intensity was lowest in location B. In contrast, the amounts of echimidine N-oxide did not follow this pattern. They were similar in locations B, C, E and F, highest in location A, and lowest in location G.

### Roots of flowering plants exhibited reduced nuclear magnetic resonance signals characteristic of sugar molecules

**Figure 8:**
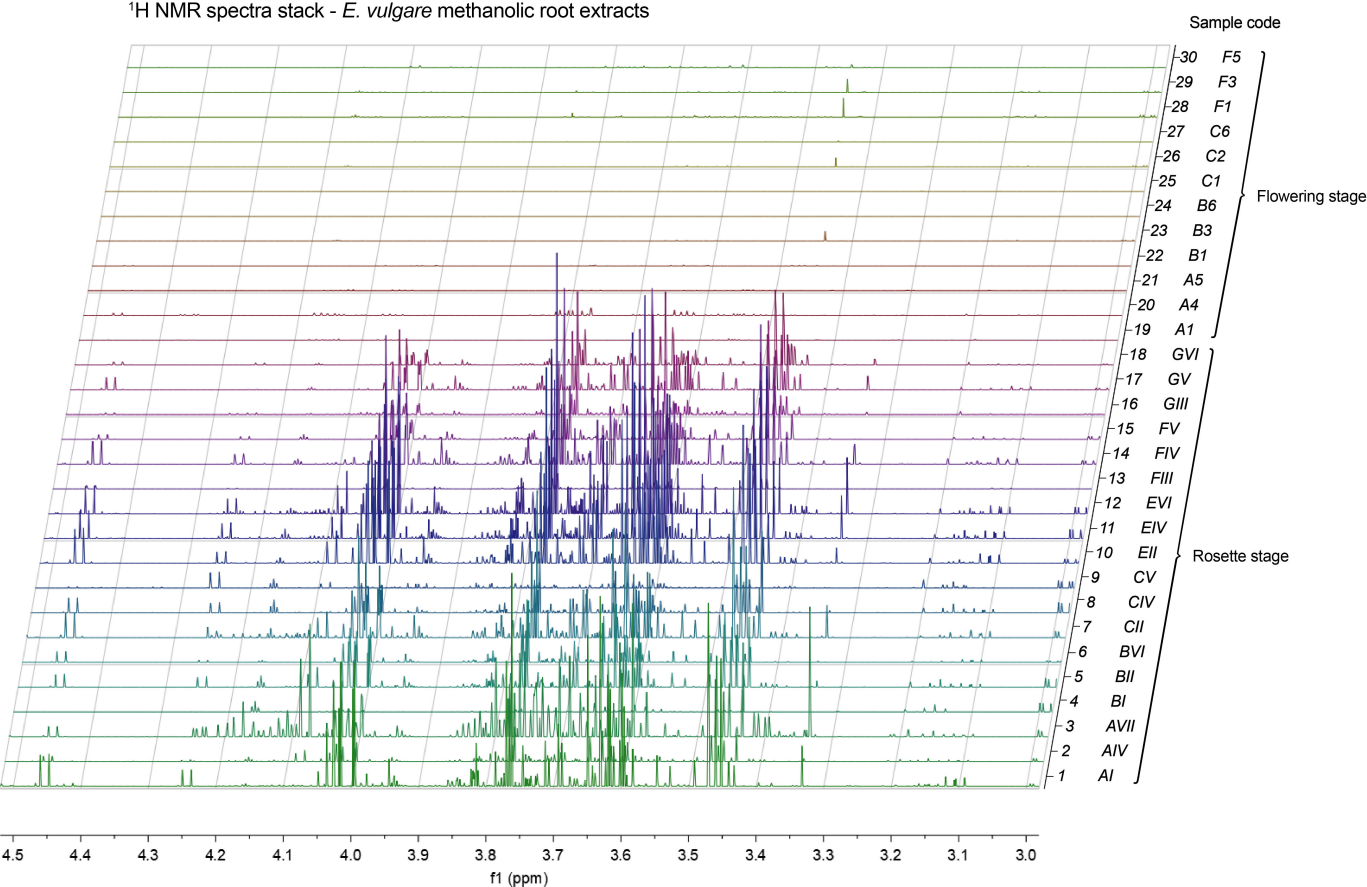
Stack of ^1^H NMR spectra of methanolic root extracts of *E. vulgare*, highlighting the 3–4.5 ppm spectral region. Spectra 1–18: rosettes, spectra 19–30: flowering plants. Sample codes are denoted to the right of each spectrum. Mnova’s resolution boosting feature used for clarity.

We employed nuclear magnetic resonance (NMR) spectroscopy to analyze selections of our samples (3 samples per sample group) with sufficient material. In the resulting spectra it was immediately obvious that the plant root extracts from flowering plants were almost entirely devoid of signals in the 3–4.5 ppm range, characteristic of sugar molecule resonances. Conversely, in the case of rosette-staged root extracts, this spectral region was populated with a multitude of overlapping complex multiplet signals that typically arise from sugars (**Figure 8**). It is of note that these signals presented the majority of the total peak area in their respective spectra, which, combined with the intrinsic quantitative nature of NMR, showed that rosette-staged plant root extracts contained a much higher total amount of metabolites than their flowering counterparts. This observation was made on our methanolic extracts, so the metabolites included in it were methanol-soluble molecules with concentrations high enough to give rise to detectable peaks in our NMR experiments. Even though we did not make metabolite annotations based on NMR spectra, it is likely that previously mentioned metabolites with sugar structural components (the annotated hexosamine(s) and N-acetylneuraminic acid) contributed to complex signal patterns in the spectra.

### Some roots of *E. vulgare* contained A/S among which ~86% were alkannins and ~14% shikonins

We measured the concentrations of 6 different A/S via HPLC. Among these, the compound of highest abundance was found to be acetyl-A/S. Out of the 65 samples analyzed, only 3 rosette-staged plant root samples, coming from 3 different locations, contained quantifiable amounts of A/S. Only these samples were hydrolyzed and subjected to normal phase chiral HPLC to determine the A/S enantiomeric ratio. The chiral analysis showed that the A/S in the samples were on average ~86% alkannins and ~14% shikonins.

### Co-occurrence network analysis identified minimal connectivity between root microbiome and metabolome of *E. vulgare* root samples

We established random forest models on normalized metabolome and root microbiome datasets to perform correlation analyses and to build co-occurrence networks based on the significant (p<0.05) strong correlations (r≥|0.7|) between metabolites and microbial rASVs. As factors, we only included location and soil pH, as these point to significant differences between groups in both datasets according to PERMANOVA together with random forest analysis and were rather balanced factors. As soil pH is a nested factor connected to the locations and the two factors resulted in highly similar networks, we only included the soil pH networks in this study, conforming the indicator taxa and rASV results of the microbiome analysis. We split the RI and RO samples to independent co-occurrence networks to separate the datasets of microorganisms which are metabolically active in the inner tissues from the ones in the outer tissue and in the remaining rhizoplane on the root surface. The RI network consisted of 21551 significant edges and 583 nodes (**Supplementary Figure 8**), with all important nodes identified as metabolites. The key vertices in the RI network were N-acetylneuraminic acid and 7-(2-methylbutyryl)-9-echimidinylretronecine. The RO network consisted of 19693 significant edges and 607 vertices, and all key nodes were metabolites. The node with maximum degree of eigenvector centrality in the RO tissue network was N-acetylneuraminic acid, maximum betweenness was at vertex 7-(2-methylbutyryl)-9-echimidinylretronecine and the key node was an unannotated metabolite (labelled M504T2 in our dataset). Notably, both networks showed major separation between the metabolic and prokaryotic clusters and the largest cliques in the networks also exclusively consisted of metabolites only, while smaller cliques consisted of ASVs. There was only one node of connection between an acidic soil pH cluster and the main metabolite cluster through a single metabolite in both networks. The metabolite labelled M504T2 (unannotated, present only in location G, under acidic soil conditions) was connected to ASV 1026 (Thermoproteota, *Nitrososphaeraceae*) and ASV 1198 (Actinomycetota*, Acidothermus*) in the RI network, while M504T2 had an edge towards ASV 1527 (Actinomycetota*, Mycobacterium*) and ASV 1296 (Actinomycetota*, Mycobacterium*) in the RO network. The closest neighboring vertices of the connected ASV 1026 and ASV 1198 in the separated acidic soil pH cliques of the RI network were ASV 1423 (Actinomycetota*, Acidothermus*), ASV 1527 (Actinomycetota, *Mycobacterium*), ASV 1296 (Actinomycetota, *Mycobacterium*), ASV 1042 (Actinomycetota, *Mycobacterium*), ASV 11 (Bacillota), ASV 1250 (Actinomycetota, *Acidothermus*) ASV 1273 (Actinomycetota, *Nakamurella*) ASV 1143 (Pseudomonadota*, Sphingomonas*) and ASV 10 (Bacillota). The closest neighbors in the soil pH clique of the RO network to ASV 1527 and ASV 1296 were ASV 1290 (Bacteroidota, *Chryseolinea*), ASV 1198 (Actinomycetota, *Acidothermus*), ASV 1423 (Actinomycetota, *Acidothermus*), ASV 1250 (Actinomycetota, *Acidothermus*), ASV 1026 (Actinomycetota, *Mycobacterium*), ASV 1143 (Pseudomonadota, *Sphingomonas*), ASV 1307 (Bacteroidota, *Chryseolinea*), ASV 1042 (Actinomycetota, *Mycobacterium*) and ASV 1516 (Actinomycetota, *Actinoplanes*).

## Discussion

We identified two distinct genetic clusters among our plant samples; however, this grouping between populations did not follow the geographic origin of the individuals, similarly to the regional findings in an *Alkanna tinctoria* L. Tausch across a bigger geographical range, where several neutral processes were responsible for the genetic structure of the plant (Ahmad et al., 2021). The influence of plant genetics on the root microbiome in *E. vulgare* could not be properly measured in our experimental setup due to imbalances and low sample numbers, however our findings in taxonomic composition and prokaryotic diversity results suggest preference of colonization by certain bacterial genera or species in plants of different genetic clusters. This host-genetic control of the microbiota was found previously in these references (Brown et al., 2020; Cordovez et al., 2021; Zhang et al., 2023).

The roots of *E. vulgare* and bulk soils obtained from the six geographical locations hosted dissimilar microbial communities. These findings are in accordance with most plant microbiome studies in which geographical distribution has been a major factor responsible for dissimilarities in plant-associated microbial community composition (Edwards et al., 2015; Escobar Rodríguez et al., 2018). The developmental stage was found to have no influence on the microbial diversity of *E. vulgare* root and bulk soil, similarly to our former greenhouse experiment on *A. tinctoria* (Csorba et al., 2022) which together with the present finding suggest a plant-family specific trait. In the present study we sampled plants at different developmental stages (vegetative rosette stage and flowering stage) but at the same timepoints, while in our previous study on *A. tinctoria* we sampled different stages in subsequent timepoints. This is contradicting *Arabidopsis thaliana* L. Heynh greenhouse studies suggesting a developmental stage-dependent microbial selection by the roots (Yuan et al., 2015) and stage-dependent rhizosphere microbiome assemblage (Chaparro et al., 2014) which can be related to the rhizoplane material sticking on the surface of our root samples. Microbiome changes related to developmental-stages were shown in other cultivated plant species: an exploratory study suggested developmental-stage driven ecological differentiation in microbial community composition in maize (Xiong et al., 2021), and the community of the rhizosphere of potato was shown to be affected by plant development as well (Pfeiffer et al., 2017).

Across six locations with contrasting soil pH, core microbiota of *E. vulgare* roots based on rASVs was identified. In this study we discovered a common core microbiome between root and soil samples, which consisted of 50 rASVs assigned to *Allorhizobium-Neorhizobium-Pararhizobium-Rhizobium*, *Cellulomonas*, *Microbacterium, Mycobacterium*, *Nocardioides*, *Pseudomonas* and *Variovorax.* The shared rASVs between soil, outer root and inner root samples (RO and RI) and the nature of the assigned species in the RO tissues allows us to speculate their presence as a result of recruitment from the soil surrounding the roots. Former studies suggest such active recruitment of microorganisms through root exudation (Karlsson et al., 2012; Hu et al., 2018; Vives-Peris et al., 2020), although our co-occurrence networks did not identify similar connections to individual root metabolites.

In line with these results, we also found rASVs from the genera *Allorhizobium-Neorhizobium-Pararhizobium-Rhizobium, Mycobacterium, Nocardioides and Sphingomonas* to be part of the root and rhizosphere core microbiome of *A. tinctoria* (Csorba et al., 2022). Albeit, the primer pair we used in our study targeting the V5-V7 region of the 16S rRNA gene is less effective for distinguishing between closely related prokaryotic species and a targeted isolation campaign would be needed to confirm the presence of these species (Beckers et al., 2016; Na et al., 2023). Besides that, only ~80% of all prokaryotic species are targeted by the primers (799F-1175R) we used, leaving a portion of prokaryotic microorganisms undiscovered (Beckers et al., 2016). Former *E. vulgare* microbiome studies were not conducted, and only one culture-independent study of the root microbiome of relatives of the family *Boraginaceae* is available for comparison.

The importance of the soil pH on the plant microbiome and metabolism is highlighted by several statistical analytical results in our study. Bacteria from the genera *Acidipila* and *Terrabacter*, and the metabolically diverse and biotechnologically important genus *Acidothermus* in root tissues and root-associated soils of *E. vulgare* correlated with low soil pH. The root samples from the acidic environment also showed a higher abundance of *Burkholderia-Caballeronia-Paraburkholderia Actinomycetospora iriomotensis* and *Mycobacterium paraterrae* which were indicator species of both root and soil samples of the moderately acidic environment. The transient core genera *Acidipila*, *Acidothermus*, *Burkholderia-Caballeronia-Paraburkholderia*, *Conexibacter*, *Crossiella*, *Cytophaga*, *Mucilaginibacter* and *Terrabacter* were found to be exclusively present under acidic soil conditions, whereas *Actinomycetospora*, *Aeromicrobium* and *Bacillus* occurred in slightly alkaline environments. pH is well known to be a major factor influencing microbial communities and several studies have shown that soil pH correlates (Tan et al., 2020) or has a strong influence on the composition of root-associated and endophytic microbial communities (ZarraonaIndia et al., 2015; Cáceres et al., 2021).

Active environmental recruitment by plant roots is an already confirmed concept (Brown et al., 2020; Zancarini et al., 2021), and it has been suggested that soil niches showing different chemical composition harbor differently composed microbial communities (Dumbrell et al., 2010). As for the metabolites, we observed increased concentrations of several amino acids, most notably glutamate and phenylalanine, in the rosette stage roots collected at the location with the lowest soil pH value. Phenylalanine, an aromatic amino acid, induces root growth (Jiao et al., 2018), is a building block of proteins but also has a significant role as a precursor to phenylpropanoids, lignin, flavonoids, anthocyanins and other plant metabolites (Pascual et al., 2016). Exogenous glutamate was found to have an inhibitory effect on primary root growth in *Arabidopsis* while stimulating branching in the primary root’s apical region (Walch-Liu et al., 2006). A recent study showed increased amino acid (glycine) uptake by maize roots under soil acidification, which was accompanied by a decrease of microbial biomass and reduced amino acid uptake by microorganisms (Pan et al., 2022). In the present study, six out of the seven amino acids (along with two dipeptides) exhibited highest abundance in *E. vulgare* roots of plants collected from a location with the lowest soil pH value, although this soil type did not have the highest nitrogen availability (**Table 1**). Other root metabolites were present in highest concentration in the location with lowest soil pH, most notably hexosamine(s). As soil pH was found to be a significant factor for both the metabolome and microbiome, we checked its influence on the associations between these two omics data.

We found significant changes in the metabolome between rosette and flowering stage plants, PAs were present in roots sampled at the rosette stage, while their abundance declined in the roots of flowering plants. One PA, echimidine, was one of the metabolites most influenced by the developmental stage. This result is in line with a study on two *Echium* species, in which *E. plantagineum* showed the highest levels of echimidine-N-oxide B and echiumine-N-oxide B in the rosette and flowering stages compared to the seedling stage (Skoneczny et al., 2015). Based on previous findings on metabolic analyses of above-ground *E. vulgare* tissues and on our finding on the decreased level of PAs in the flowering stage, it might be that PAs are initially synthesized in the roots and then transported to the aerial parts. Several studies isolated and identified *Echium* PAs in the plant´s pollen (Boppré et al., 2005; Beales et al., 2007; Cairns et al., 2015; Kast et al., 2018) and nectar (Lucchetti et al., 2016). These compounds in the roots could serve as defense against herbivores and *Echium* pests, such as the common oligophage of *E. vulgare Mogulones geographicus* (Gosik, 2010), however their effects on microorganisms and microbial pathogens are unknown. The PA compound 7-(2-methylbutyryl)-9-echimidinylretronecine was not previously reported in other *Echium* metabolic studies. This compound, previously reported in the leaves of endemic capeverdian *Echium* species (Carvalho et al., 2013), eluted mere seconds after echimidine, to which it is very similar structurally, but only a tentative annotation was performed, due to the lack of availability of MS/MS fragmentation spectra in databases. It is thus necessary to confirm the presence of this PA in future studies. Random forest and correlation analyses highlighted a hexosamine (or multiple hexosamines, as we could not differentiate between them) as a metabolite highly associated with the developmental stage. This metabolite was almost absent in the roots at flowering stage across samples from all locations. Our NMR analysis suggested that this concentration trend held for other sugars as well. In the rosette stage samples, the spectral region in which sugar resonances are typically found was populated by a number of complex multiplet signals and was indeed the spectral region with the largest amount of signals, both in terms of number and intensity/peak area. Sugars (e.g., sucrose) promote flowering in plants, as confirmed by an experiment on *Sinapis alba*, where exogenous sucrose was found to induce flowering (Bernier et al., 1993), or by a study on grapevine where sugars were mobilized from roots, trunks and canes for flower formation (Lebon et al., 2008). Due to our focus on roots, it was not possible to determine the fate of sugars from the rosette stage in the aerial part of *E. vulgare*, nor their potential role in regulating the timing of flowering. Even though our study suggests a near-depletion of sugars through the rosette-flowering stage transition and therefore a key role these energy molecules play in *E. vulgare* flowering, further studies involving the entirety of the plant are necessary to evaluate their potential role in regulating flowering timing. Furthermore, while we found the medicinal compounds alkannin and shikonin in the roots of the collected *E. vulgare* plants, their concentrations were not consistent between samples from the same locations, unlike in other typical producers of A/S (Tappeiner et al., 2014). This observed difference might be due to an underlying genetic or environmental factor in *E. vulgare* plants.

Co-occurrence network analyses based on soil pH in the RI and RO tissues showed equally low connectivity between metabolites and microorganisms and some clustering according to soil pH categories. In the limited connections between microbiome and metabolome in the soil pH co-occurrence networks, unannotated compounds correlated and supposedly interacted with microbial taxa of *Nitrososphaeraceae*, *Acidothermus* and *Mycobacterium*. The current literature lacks comprehensive studies or investigations addressing the interconnected relationship between soil pH, plant metabolome, and the plant microbiome which could be used to confirm or contradict our findings. In a study a pathogen infection affected the connectivity and correlation of only a handful of metabolome-microbiome connections in a reduced network (Kudjordjie et al., 2022). Our previous study on *A. tinctoria* grown in the greenhouse showed a wider connectivity between metabolites and bacterial and fungal rASVs, and a subnetwork of only six identified metabolites clearly linked the production of some important metabolites to the presence of bacteria and fungi (Csorba et al., 2022).

Our initial hypothesis regarding a diverse microbiome and metabolome across different geographical locations and environmental factors was validated, particularly with soil pH identified as a significant influencing parameter. Additionally, it was observed that plant developmental stages exclusively affected the metabolic composition of *E. vulgare* roots, while some dissimilarities in microbiome composition were attributed to plant genetic clustering. The varying impact of the investigated environmental factors on the plant microbiome and metabolome furthermore features the importance of multi-omics studies and widens the concept of environmental impact on complex biological systems.

## Data availability statement

The datasets presented in this study can be found in online repositories. The names of the repository/repositories and accession number(s) can be found below: https://www.ncbi.nlm.nih.gov/, PRJNA1010776 https://www.metabolomicsworkbench.org/data/, project ID: PR001283.

## Author contributions

CC: Conceptualization, Data curation, Formal analysis, Investigation, Methodology, Resources, Software, Validation, Visualization, Writing – original draft, Writing – review & editing. NR: Data curation, Formal analysis, Investigation, Methodology, Software, Validation, Visualization, Writing – original draft, Writing – review & editing. LA: Data curation, Formal analysis, Methodology, Software, Writing – review & editing. AS: Conceptualization, Funding acquisition, Project administration, Supervision, Writing – review & editing. AV: Formal analysis, Investigation, Methodology, Writing – original draft. MA: Formal analysis, Investigation, Methodology, Visualization, Writing – original draft. SC: Investigation, Methodology, Visualization, Writing – original draft. MP: Methodology, Supervision, Writing – review & editing. EM: Conceptualization, Methodology, Supervision, Writing – review & editing. AA: Conceptualization, Funding acquisition, Project administration, Supervision, Writing – review & editing. GB: Conceptualization, Funding acquisition, Methodology, Project administration, Resources, Supervision, Writing – review & editing.
