# Supplementary material for: Soil pH, developmental stages and geographical origin differently influence the root metabolomic diversity and root-related microbial diversity of Echium vulgare from native habitats
Source: Front Plant Sci. 2024 Jun 24;15:1369754. doi: 10.3389/fpls.2024.1369754 (PMC11232435; doi:10.3389/fpls.2024.1369754)
Supplement: Supplementary file 1 [file DataSheet_1.docx]

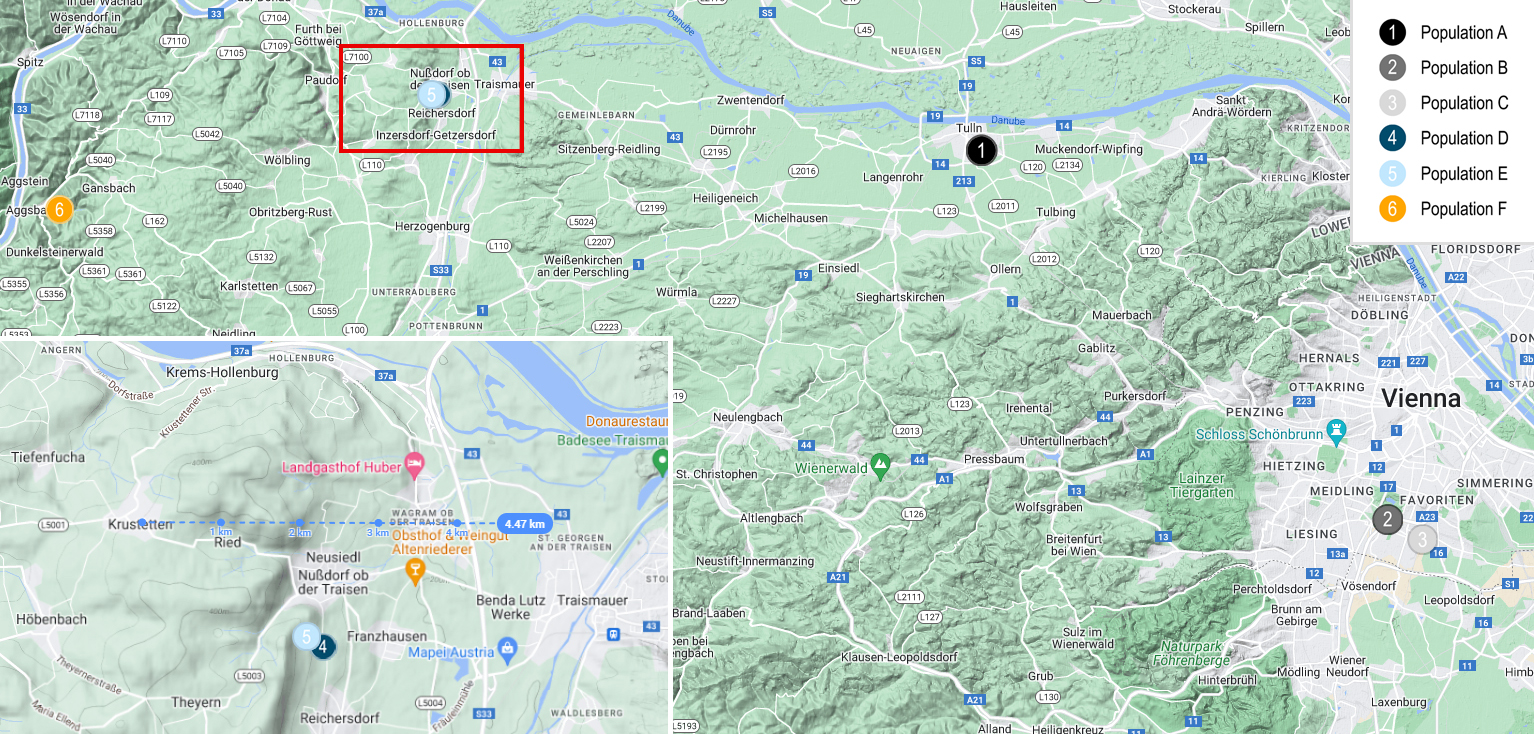


Supplementary Figure 1 Map of the locations based on the collected GPS coordinates (created with Google Maps). Location 4 and 5 highlighted in a separate box due to their small distance.


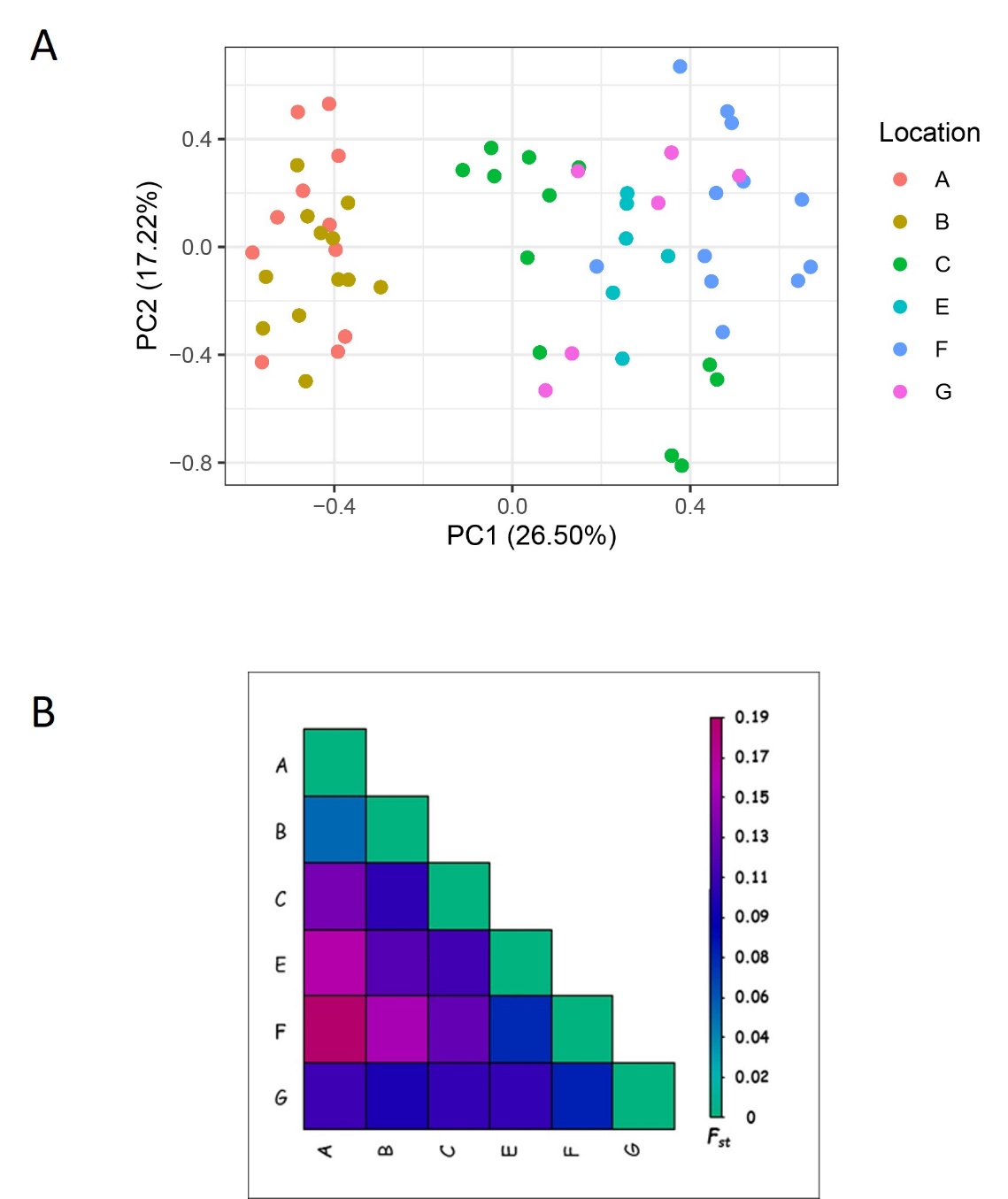


Supplementary Figure 2 **A** Principal coordinate analysis (PCoA) based on Bruvo genetic distances between 60 individuals of Echium vulgare based on microsatellite marker analysis collected from wild. The individuals are colored based on their geographic locations. **B** Pairwise genetic distances among 6 populations of Echium vulgare


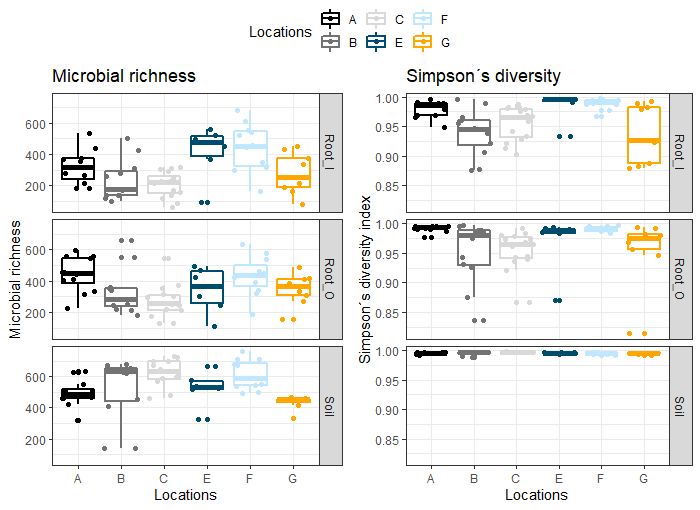


Supplementary Figure 3 Boxplots of microbial richness and Simpson´s diversity in the different locations, faceted by the 3 sample types. Grey-scale colors are the locations with soils with neutral pH, blue colored locations had slightly acidic pH and the yellow color represents the moderately acidic soil conditions.


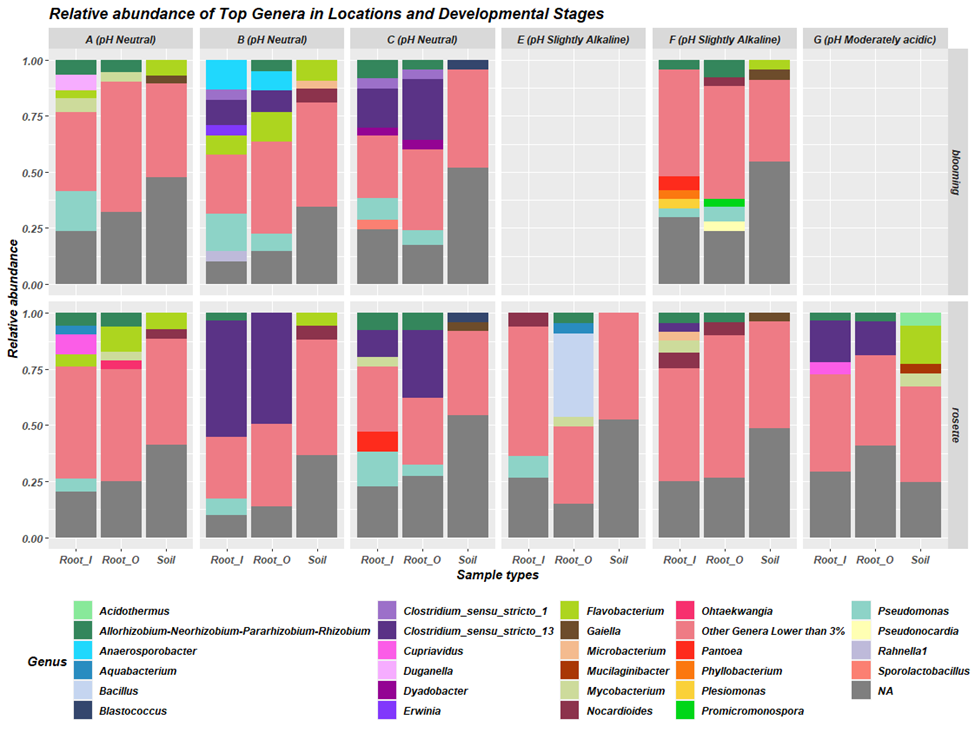


Supplementary Figure 4 Relative abundance of top Genera faceted by developmental stages and locations with the corresponding pH categories.


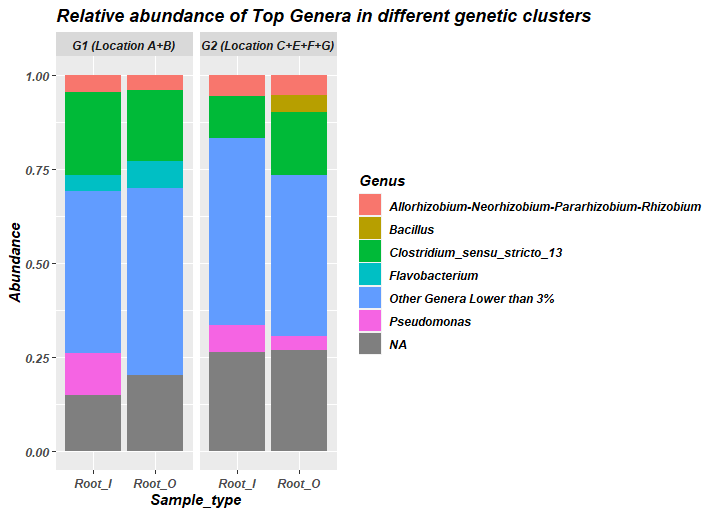


Supplementary Figure 5 Barplot of relative abundance of top genera (>5%) in different genetic clusters


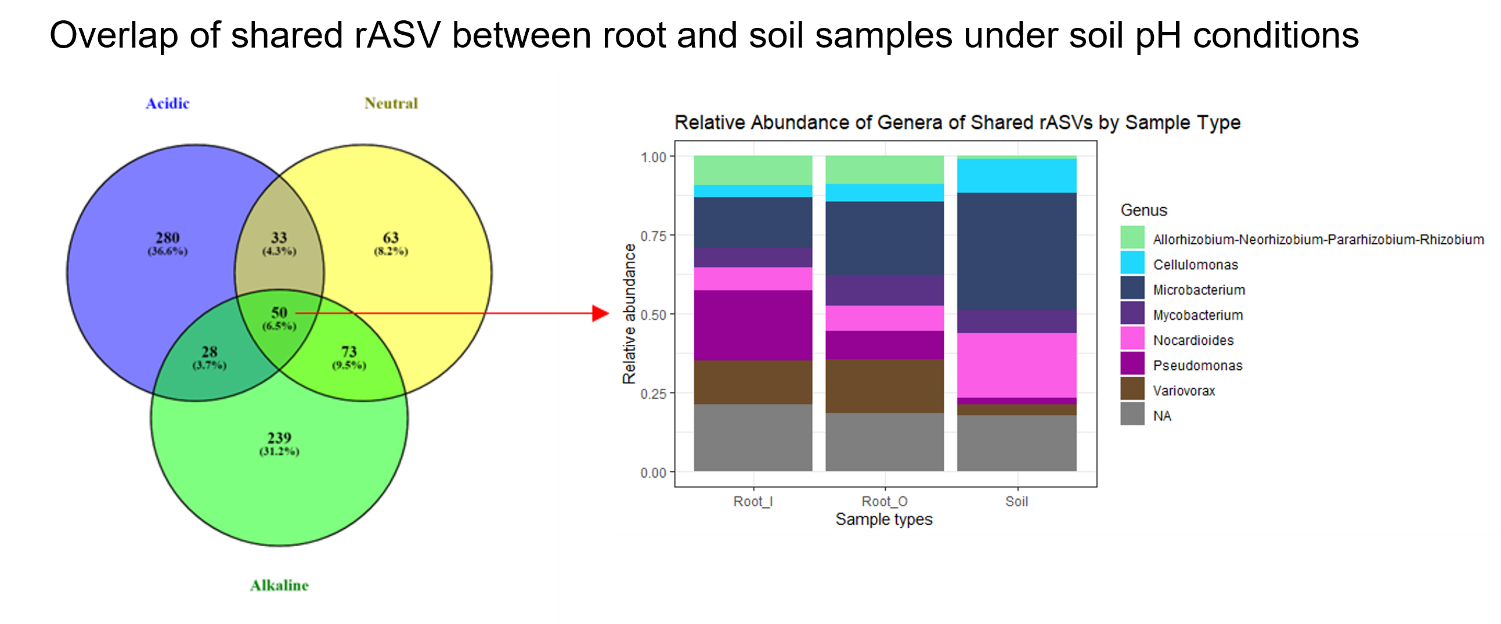


Supplementary Figure 6 Venn diagram of shared rASVs between all samples and relative abundance of shared rASVs by sample type

*
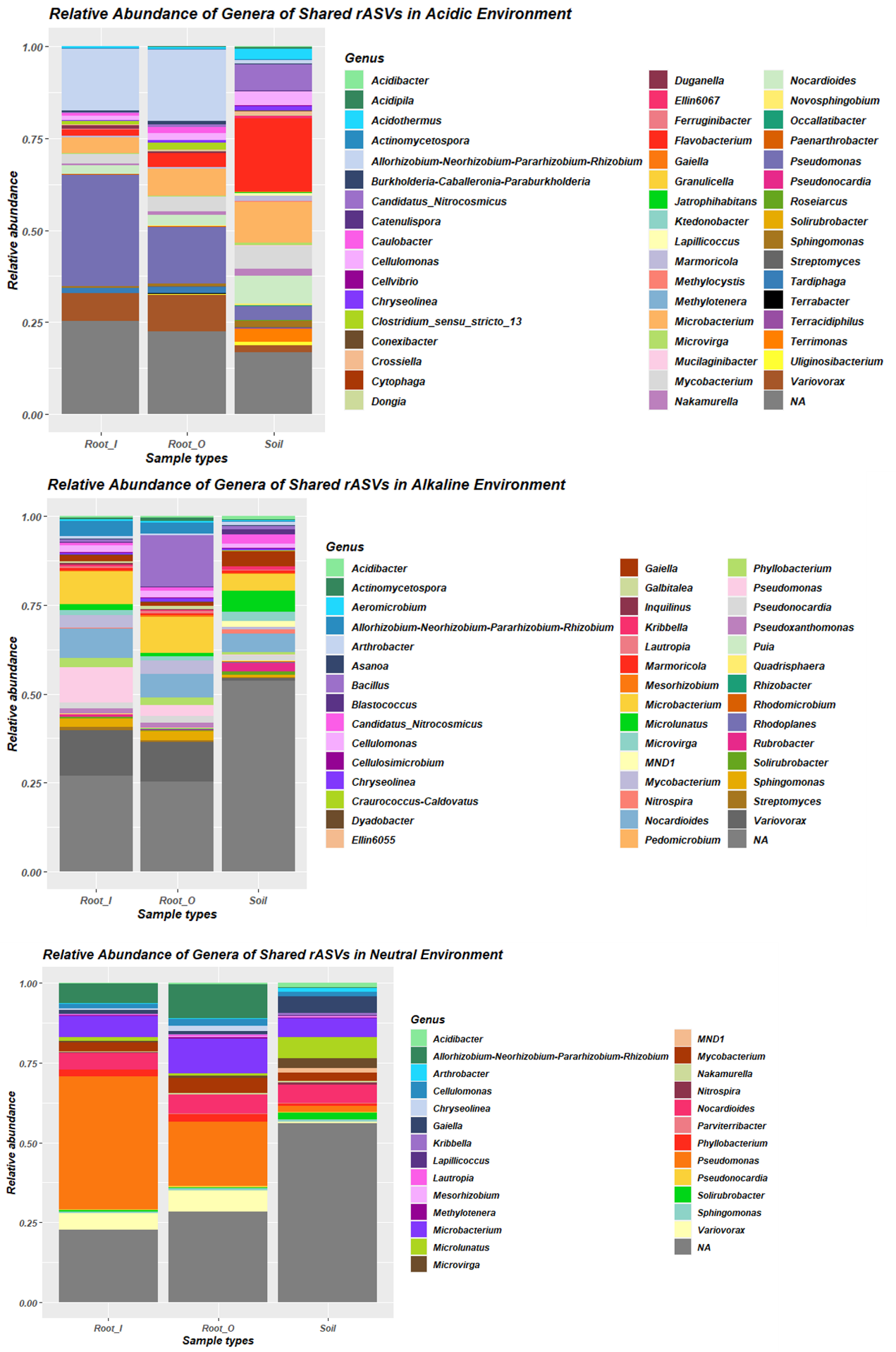
*

**Supplementary Figure 7** Relative abundance of transient core microbiota rASVs shared between root and soil samples in different soil pH conditions


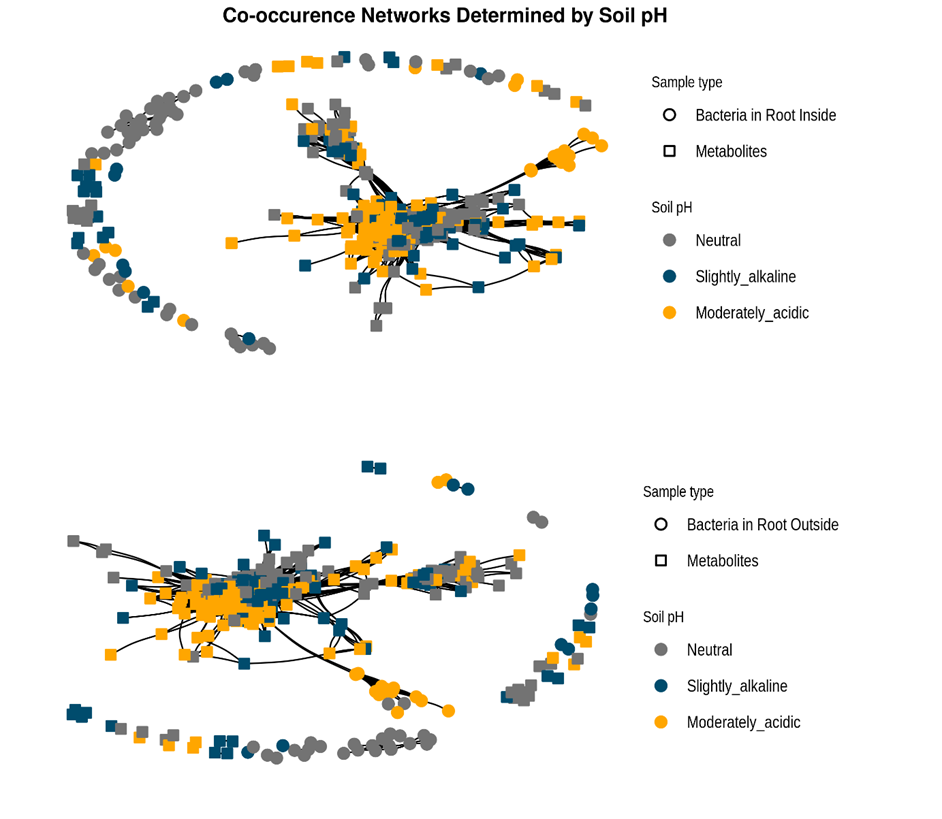


Supplementary Figure 8 Co-occurrence networks based on significant strong correlations (r>0.7) determined by soil pH in Root Inside samples and Root Outside samples. Colors represent the soil pH categories, while shapes show prokaryotic rASVs or metabolites from the untargeted LC-MS analysis.

Supplementary Table 1 Description, coordinates and EUNIS habitat classification code of the six sampling locations

| **Location** | **Description** | **GPS coordinate** | **Area type** | **EUNIS code** |
| --- | --- | --- | --- | --- |
| **A** | UFT Tulln | 48°19'15.2"N 16°04'10.9"E | lawn, partly next to Prunus shrubs, semi-ruderal, flat area | J2 |
| **B** | Anton Balzer Weg, 1100 Wien | 48°09'10.8"N 16°20'53.9"E | ruderal meadow, East exposed | J2 |
| **C** | Bahndamm, Pottendorfer Linie, 1230 Wien | 48°08'37.3"N 16°22'17.0"E | ruderal meadow, West exposed, next to railway, | J4 |
| **E** | between Reichersdorf and Nußdorf ob der Traisen, E exposed | 48°20'47.8"N 15°41'43.8"E | loess, next to grapevine yard, E exposed | I2 |
| **F** | between Reichersdorf and Nußdorf ob der Traisen, S exposed | 48°20'51.7"N 15°41'34.4"E | loess, next to grapevine yard, S exposed | I2 |
| **G** | ca 1km east of Aggsbach Dorf | 48°17'40.6"N 15°26'09.6"E | gravel rich soil next to animal grazing place | I2 |

Supplementary Table 2 Pairwise Permanova on CSS normalized table based on Bray-Curtis distances with 999 permutations. In sample subsets location pairs, soil pH categories and developmental stages were analysed. Neut.= neutral, Sl.Alk.=slightly alkaline, Mod.Ac.=moderately acidic. P values are false discovery rate adjusted.

| All samples | | | | | | | | |
| --- | --- | --- | --- | --- | --- | --- | --- | --- |
| Pairs | | **F.Model** | | **R2** | | **P value** | | |
| Root inside vs Root outside | | 0.5846 | | 0.0045 | | 0.9820 | | |
| Soil vs Root inside | | 6.4947 | | 0.0494 | | **0.0015**** | | |
| Soil vs Root outside | | 6.7525 | | 0.0516 | | **0.0015**** | | |
| Root inside samples | | | | **Root outside samples** | | | | |
| Pairs | **F.Model** | **R^2^** | **P value** | **Pairs** | **F.Model** | | **R2** | **P value** |
| A vs B | 1.6427 | 0.0725 | **0.0230*** | **A vs B** | 2.3087 | | 0.0949 | **0.0038**** |
| A vs C | 2.2082 | 0.0812 | **0.0033**** | **A vs C** | 2.3467 | | 0.0858 | **0.0075**** |
| A vs E | 3.7717 | 0.2009 | **0.0019**** | **A vs E** | 4.3754 | | 0.0858 | **0.0021**** |
| A vs F | 8.0313 | 0.2766 | **0.0019**** | **A vs F** | 8.0663 | | 0.2775 | **0.0021**** |
| A vs G | 3.7410 | 0.1721 | **0.0019**** | **A vs G** | 4.2337 | | 0.1822 | **0.0021**** |
| B vs C | 2.1109 | 0.0751 | **0.0123*** | **B vs C** | 1.7602 | | 0.0658 | **0.0321*** |
| B vs E | 4.4626 | 0.2181 | **0.0019**** | **B vs E** | 4.7885 | | 0.2303 | **0.0021**** |
| B vs F | 8.5797 | 0.2806 | **0.0019**** | **B vs F** | 7.5629 | | 0.2648 | **0.0021**** |
| B vs G | 4.6029 | 0.1950 | **0.0019**** | **B vs G** | 4.7566 | | 0.2002 | **0.0021**** |
| C vs E | 2.5451 | 0.1129 | **0.012*** | **C vs E** | 2.2533 | | 0.1060 | **0.0200*** |
| C vs F | 4.8696 | 0.1577 | **0.0019**** | **C vs F** | 3.9220 | | 0.1405 | **0.0021**** |
| C vs G | 2.7785 | 0.1078 | **0.0019**** | **C vs G** | 2.6955 | | 0.1091 | **0.005**** |
| E vs F | 1.4621 | 0.0837 | 0.1136 | **E vs F** | 1.9199 | | 0.1135 | **0.0312*** |
| E vs G | 1.5435 | 0.1061 | 0.121 | **E vs G** | 2.1661 | | 0.1429 | **0.0390*** |
| F vs G | 2.5832 | 0.1197 | **0.0125*** | **F vs G** | 2.8599 | | 0.1371 | **0.0081**** |
| Neut. vs Sl.Alk. | 9.3220 | 0.1449 | **0.0015**** | **Neut. vs Sl.Alk.** | 8.3807 | | 0.1343 | **0.0015**** |
| Neut. vs Mod. Ac. | 4.052721 | 0.0809 | **0.0015**** | **Neut vs Mod.Ac.** | 4.2771 | | 0.0851 | **0.0015**** |
| Sl.Alk. vs Mod.Ac. | 2.6193 | 0.0948 | **0.0080**** | **Sl.Alk. vs Mod.Ac.** | 3.0770 | | 0.1136 | **0.0030**** |
| Rosette vs Blooming | 1.0862 | 0.0167 | 0.317 | **Rosette vs Blooming** | 1.3236 | | 0.0206 | 0.141 |
| Bulk Soil | | | | | | | | |
| Pairs | **F Model** | **R^2^** | **P value** | **Pairs** | **F Model** | | **R^2^** | **P value** |
| A vs B | 2.3087 | 0.9497 | **0.0019**** | **Neut. vs Sl.Alk.** | 8.3807 | | 0.1343 | **0.0015**** |
| A vs C | 2.3467 | 0.0858 | **0.0082**** | **Neut. vs Mod. Ac.** | 4.2772 | | 0.0851 | **0.0015**** |
| A vs E | 4.3754 | 0.2147 | **0.0019**** | **Sl.Alk. vs Mod.Ac.** | 3.0769 | | 0.1136 | **0.0050**** |
| A vs F | 8.0662 | 0.2775 | **0.0019**** | **Rosette vs Blooming** | 1.3236 | | 0.0205 | 0.128 |
| A vs G | 4.2336 | 0.1822 | **0.0019**** |  |  |  |  |  |
| B vs C | 1.7602 | 0.0658 | **0.0300*** |  |  |  |  |  |
| B vs E | 4.7885 | 0.2303 | **0.0019**** |  |  |  |  |  |
| B vs F | 7.5629 | 0.2647 | **0.0019**** |  |  |  |  |  |
| B vs G | 4.7566 | 0.2002 | **0.0019**** |  |  |  |  |  |
| C vs E | 2.2533 | 0.1060 | **0.0196*** |  |  |  |  |  |
| C vs F | 3.9220 | 0.1405 | **0.0019**** |  |  |  |  |  |
| C vs G | 2.6955 | 0.1091 | **0.0075**** |  |  |  |  |  |
| E vs F | 1.9199 | 0.1135 | **0.0246*** |  |  |  |  |  |
| E vs G | 2.1661 | 0.1428 | **0.0196*** |  |  |  |  |  |
| F vs G | 2.8599 | 0.1371 | **0.0075**** |  |  |  |  |  |

^a^statistically significant p values presented in bold letters, *p<0.05, **p<0.01

Supplementary Table 3 Regression analysis results on Alpha diversity measures. Richness and Simpson´s diversity combined.

| Sample type | Factor | %IncMSE | P value | IncNodePurity | P value |
| --- | --- | --- | --- | --- | --- |
| Inner root | Soil pH | 30.4486 | 0.001** | 226229.62 | 0.001** |
|  | Location | 24.8646 | 0.005** | 228573.23 | 0.002** |
|  | Developmental stage | -0.6200 | 0.379 | 38631.17 | 0.499 |
| Outer root | Soil pH | 15.0470 | 0.044* | 60469.86 | 0.085 |
|  | Location | 27.7162 | 0.005** | 168661.52 | 0.006** |
|  | Developmental stage | -0.819 | 0.310 | 24920.96 | 0.758 |
| Soil | Soil pH | 20.3391 | 0.002* | 65539.80 | 0.021* |
|  | Location | 25.6375 | 0.007* | 139297 | 0.011 |
|  | Developmental stage | -10.6069 | 0.695 | 19755.66 | 0.924 |

Supplementary Table 4 Fold changes of selected PAs between the rosette and flowering stages, across different locations. The t-test p-values for the individual metabolic features are listed in the brackets for statistically significant metabolites.

| Metabolite | Fold change (t-test p-value) | | | |
| --- | --- | --- | --- | --- |
|  | **Location A** | **Location B** | **Location C** | **Location F** |
| 7-(2-Methylbutyryl)-9-echimidinylretronecine | 0.15 (p<0.01) | 0.23 (p<0.05) | ≥0.5 | 0.06 (p<0.01) |
| 7-(2-Methylbutyryl)-9-echimidinylretronecine N-oxide | ≥0.5 | ≥0.5 | ≥0.5 | ≥0.5 |
| 9-angeloyltrachelamthamidine | ≥0.5 | 0.01 (p<0.01) | 0.01 (p<0.01) | ≥0.5 |
| Acetylechimidine | 0.06 (p<0.01) | 0.02 (p<0.01) | 0.001 (p<0.01) | 0.05 (p<0.01) |
| Acetylechimidine N-oxide | 0.14 (p<0.01) | 0.004 (p<0.05) | 0.001 (p<0.01) | 0.07 (p<0.05) |
| Echimidine | 0.06 (p<0.01) | 0.01 (p<0.01) | 0.003 (p<0.01) | 0.09 (p<0.01) |
| Echimidine N-oxide | 0.25 (p<0.01) | ≥0.5 | 0.005 (p<0.01) | ≥0.5 |
| Echiuplatine | 0.10 (p=0.01) | 0.02 (p<0.01) | 0.005 (p<0.01) | 0.02 (p<0.01) |
| Heliocurassavicine N-oxide | ≥0.5 | 0.29 (p<0.05) | 0.22 (p<0.01) | ≥0.5 |
| Intermedine N-oxide | ≥0.5 | ≥0.5 | 0.14 (p<0.01) | ≥0.5 |
| Leptanthine N-oxide / Echimiplatine N-oxide | ≥0.5 | ≥0.5 | 0.02 (p<0.01) | ≥0.5 |
| Uplandicine | 0.04 (p<0.01) | 0.12 (p=0.01) | 0.02 (p<0.01) | ≥0.5 |
| Uplandicine N-oxide | 0.11 (p<0.01) | ≥0.5 | 0.003 (p<0.01) | ≥0.5 |
| Viridiflorine | ≥0.5 | 0.26 (p<0.05) | 0.15 (p<0.01) | ≥0.5 |

*Supplementary Table 5. Details on parent and fragment ions used for metabolite annotation.*

| Compound | Chemical formula | ID method | Retention time [min] | Parent ion mass [m/z] | Parent ion type | Theoretical ion mass [m/z] | Δm [ppm] | Matched fragments (rel. int. >10%) | Source |
| --- | --- | --- | --- | --- | --- | --- | --- | --- | --- |
| 2-Aminobutyric acid | C_4_H_9_NO_2_ | MS/MS (+) | 0.9 | 104.0700 | [M+H]^+^ | 104.0706 | -5.77 | 86.8, 59.8 | PubChem |
| 7-(2-Methylbutyryl)-9-echimidinylretronecine | C_20_H_33_NO_7_ | MS/MS (+) | 3.3 | 400.2312 | [M+H]^+^ | 400.2330 | -4.50 | 222.1, 221.5, 120.9, 119.9 | El-Shazly *et al.*, 2014 |
| 7-(2-Methylbutyryl)-9-echimidinylretronecine N-oxide | C_20_H_33_NO_8_ | MS (+) | 3.3 | 416.2261 | [M+H]^+^ | 416.2279 | -4.32 | NA | NA |
| 9-Angeloyltrachelanthamidine | C_13_H_21_NO_2_ | MS/MS (+) | 3.1 | 224.1634 | [M+H]^+^ | 224.1645 | -4.91 | 139.9, 123.9, 121.9, 82.8 | El-Shazly *et al.*, 2014 |
| Acetylalkannin | C_18_H_18_O_6_ | MS/MS (+), HPLC | 8.2 | 353.0988 | [M+Na]^+^ | 353.0996 | -2.27 | 352.8, 345.7, 342.4, 335.2, 328.3, 311.1, 293.0, 262.1, 253.0, 183.1, 111.9 | Standard |
| Acetylechimidine | C_22_H_33_NO_8_ | MS/MS (+) | 3.4 | 440.2258 | [M+H]^+^ | 440.2279 | -4.77 | 422.3, 380.3, 336.2, 322.1, 220.0 | Betteridge *et al.*, 2005 |
| Acetylechimidine N-oxide | C_22_H_33_NO_9_ | MS/MS (+) | 3.4 | 456.2205 | [M+H]^+^ | 456.2228 | -5.04 | 438.2, 396.2, 352.2, 338.1, 254.1 | Betteridge *et al.*, 2005 |
| Alanine | C_3_H_7_NO_2_ | MS/MS (+) | 0.9 | 90.0542 | [M+H]^+^ | 90.0550 | -8.88 | 71.7 | mzCloud |
| Alanylproline | C_8_H_14_N_2_O_3_ | MS/MS (+) | 1.5 | 187.1067 | [M+H]^+^ | 187.1077 | -5.34 | 168.8, 115.9 | mzCloud |
| Alkannin | C_16_H_16_O_5_ | MS/MS (+), HPLC | 7.4 | 289.1057 | [M+H]^+^ | 289.1071 | -4.84 | 271.0 | Standard |
| Anthranilic acid | C_7_H_7_NO_2_ | MS/MS (+) | 2.4 | 138.0544 | [M+H]^+^ | 138.0550 | -4.35 | 119.8, 93.8 | mzCloud |
| Arachidonic acid | C_20_H_32_O_2_ | MS/MS (+) | 10.6 | 305.2462 | [M+H]^+^ | 305.2475 | -4.26 | 259.1, 231.2, 120.9 | HMDB, PubChem |
| Arginine | C_6_H_14_N_4_O_2_ | MS/MS (+) | 0.8 | 175.1178 | [M+H]^+^ | 175.1190 | -6.85 | 158.0, 156.9, 129.9, 115.9, 111.9, 69.9, 59.8 | mzCloud |
| Echimidine | C_20_H_31_NO_7_ | MS/MS (+) | 3.2 | 398.2157 | [M+H]^+^ | 398.2173 | -4.02 | 380.1, 336.1, 220.0, 119.9 | Betteridge *et al.*, 2005 |
| Echimidine N-oxide | C_20_H_31_NO_8_ | MS/MS (+) | 3.1 | 414.2102 | [M+H]^+^ | 414.2122 | -5.55 | 396.2, 356.3, 352.2, 338.1, 254.1 | Betteridge *et al.*, 2005 |
| Echiuplatine | C_20_H_31_NO_6_ | MS/MS (+) | 3.6 | 382.2214 | [M+H]^+^ | 382.2224 | -2.62 | 220.0, 119.9 | Betteridge *et al.*, 2005 |
| Glutamate | C_5_H_9_NO_4_ | MS/MS (+) | 1.0 | 148.0597 | [M+H]^+^ | 148.0604 | -4.73 | 129.8, 101.8, 83.8 | mzCloud, GNPS |
| Heliocurassavicine N-oxide | C_15_H_27_NO_5_ | MS/MS (+) | 2.7 | 302.1948 | [M+H]^+^ | 302.1962 | -4.63 | 284.1, 258.1, 158.0, 139.9 | GNPS |
| Hexosamine | C_6_H_13_NO_5_ | MS/MS (+) | 0.9 | 162.0752 | [M-H_2_O+H]^+^ | 162.0761 | -5.55 | 144.8, 143.9, 126.9, 125.8, 113.8, 101.8, 97.9, 95.9, 84.8, 83.8, 71.9, 59.9 | GNPS |
| Intermedine N-oxide | C_15_H_25_NO_6_ | MS/MS (+) | 2.7 | 316.1749 | [M+H]^+^ | 316.1755 | -1.90 | 272.1, 226.1, 172.0, 154.9, 138.0, 135.9 | GNPS |
| Intermedine/Lycopsamine | C_15_H_25_NO_5_ | MS/MS (+) | 2.6 | 300.1798 | [M+H]^+^ | 300.1805 | -2.33 | 155.9, 137.9, 119.9, 93.8 | Shazly *et al.*, 2014; GNPS |
| Isovalerylalkannin | C_21_H_24_O_6_ | MS/MS (+), HPLC | 9.7 | 395.1443 | [M+Na]^+^ | 395.1465 | -5.57 | 409.3, 394.2, 377.2, 345.7, 344.8, 343.8, 343.2, 342.3, 341.1, 340.2, 339.2, 328.7, 327.9, 326.7, 292.9 | Standard |
| Leptanthine | C_15_H_25_NO_6_ | MS/MS (+) | 2.3 | 316.1740 | [M+H]^+^ | 316.1755 | -4.74 | 254.0, 137.9, 120.1, 93.9 | Shazly *et al.*, 2014 |
| Leptanthine N-oxide/Echimiplatine N-oxide | C_15_H_25_NO_7_ | MS/MS (+) | 2.4 | 332.1694 | [M+H]^+^ | 332.1704 | -3.01 | 314.1, 270.0, 256.1, 228.1, 172.0 | Betteridge *et al*. |
| Leucine | C_6_H_13_NO_2_ | MS/MS (+) | 2.2 | 132.1013 | [M+H]^+^ | 132.1019 | -4.54 | 85.9 | mzCloud, GNPS |
| Methyl dihydrojasmonate | C_13_H_22_O_3_ | MS/MS (+) | 5.7 | 244.1896 | [M+NH_4_]^+^ | 244.1907 | -4.50 | 226.1, 225.3, 212.1 | PubChem |
| N-acetylneuraminic acid | C_11_H_19_NO_9_ | MS/MS (+) | 1.6 | 292.1011 | [M-H_2_O+H]^+^ | 292.1027 | -5.48 | 274.1 | MoNA, mummichog |
| Ornithine | C_5_H_12_N_2_O_2_ | MS (+) | 1.4 | 118.0858 | [M-NH_3_+H]^+^ | 118.0863 | -4.23 | NA | XCMS Online/mummichog |
| Phenylalanine | C_9_H_11_NO_2_ | MS/MS (+) | 2.5 | 166.0853 | [M+H]^+^ | 166.0863 | -6.02 | 119.9 | mzCloud, GNPS |
| Proline | C_5_H_9_NO_2_ | MS/MS (+) | 1.0 | 116.0700 | [M+H]^+^ | 116.0706 | -5.17 | 69.8 | mzCloud, GNPS |
| Threonine | C_4_H_9_NO_3_ | MS/MS (+) | 0.9 | 120.0648 | [M+H]^+^ | 120.0655 | -5.83 | 101.9, 73.8 | mzCloud, GNPS |
| Uplandicine | C_17_H_27_NO_7_ | MS/MS (+) | 2.5 | 358.1855 | [M+H]^+^ | 358.1860 | -1.40 | 179.9, 119.9 | El-Shazly *et al.*, 2014 |
| Uplandicine N-oxide | C_17_H_27_NO_8_ | MS/MS (+) | 2.6 | 374.1789 | [M+H]^+^ | 374.1809 | -5.35 | 356.1, 312.1, 298.2, 214.0 | Betteridge *et al.*, 2005 |
| Valine | C_5_H_11_NO_2_ | MS/MS (+) | 1.4 | 118.0858 | [M+H]^+^ | 118.0863 | -4.23 | 71.9 | mzCloud, GNPS |
| Valylproline | C_10_H_18_N_2_O_3_ | MS/MS (+) | 2.3 | 215.1379 | [M+H]^+^ | 215.1390 | -5.11 | 116.0 | mzCloud |
| Viridiflorine | C_15_H_27_NO_4_ | MS/MS (+) | 2.8 | 286.1999 | [M+H]^+^ | 286.2013 | -4.89 | 141.9, 123.9 | El-Shazly *et al.*, 2014 |
| β, β - dimethylacrylalkannin | C_21_H_22_O_6_ | MS/MS (+), HPLC | 9.5 | 393.1288 | [M+Na]^+^ | 393.1309 | -5.34 | 392.7, 392.1, 391.4, 375.1, 351.0, 345.5, 344.8, 344.0, 342.7, 341.3, 340.2, 328.4, 293.3, 253.1 | Standard |
